# Comparison of Subjective and Objective Methods to Measure the Physical Activity of Non-Depressed Middle-Aged Healthy Subjects with Normal Cognitive Function and Mild Cognitive Impairment—A Cross-Sectional Study

**DOI:** 10.3390/ijerph18158042

**Published:** 2021-07-29

**Authors:** Aleksandra Makarewicz, Małgorzata Jamka, Maria Wasiewicz-Gajdzis, Joanna Bajerska, Anna Miśkiewicz-Chotnicka, Jarosław Kwiecień, Aleksandra Lisowska, Dominque Gagnon, Karl-Heinz Herzig, Edyta Mądry, Jarosław Walkowiak

**Affiliations:** 1 Department of Pediatric Gastroenterology and Metabolic Diseases, Poznan University of Medical Sciences, 27/33 Szpitalna Str., 60-572 Poznań, Poland; a.u.makarewicz@gmail.com (A.M.); mjamka@ump.edu.pl (M.J.); marija.wa@gmail.com (M.W.-G.); chotnicka@ump.edu.pl (A.M.-C.); karl-heinz.herzig@oulu.fi (K.-H.H.); 2 Department of Human Nutrition and Dietetics, Poznan University of Life Sciences, 31 Wojska Polskiego Str., 60-624 Poznań, Poland; joanna.bajerska@up.poznan.pl; 3 Department of Pediatrics, Faculty of Medical Sciences in Zabrze, Medical University of Silesia in Katowice, 15-3 Maja Str., 41-800 Zabrze, Poland; jkwiecien@sum.edu.pl; 4 Department of Clinical Auxology and Pediatric Nursing, Poznan University of Medical Sciences, 27/33 Szpitalna Str., 60-572 Poznań, Poland; alisowska@ump.edu.pl; 5 Medical Research Center, Oulu University Hospital, University of Oulu, 50 Kajaanintie Str., 90220 Oulu, Finland; dominique.gagnon@oulu.fi; 6 Research Unit of Biomedicine, University of Oulu, 1 Pentti Kaiteran Ktu Str., 90220 Oulu, Finland; 7 Department of Physiology, Poznan University of Medical Sciences, 6 Święcickiego Str., 60-781 Poznań, Poland; emadry@ump.edu.pl

**Keywords:** accelerometry, physical activity, questionnaire, self-report, cognition, Montreal Cognitive Assessment

## Abstract

This study compared subjective and objective methods of measuring different categories of physical activity in non-depressed middle-aged subjects with normal cognitive function (NCF) and mild cognitive impairment (MCI). In total, 75 participants (NCF: *n* = 48, MCI: *n* = 27) were recruited and physical activity was assessed for seven days using the ActiGraph and the International Physical Activity Questionnaire (IPAQ). Anthropometric parameters, body compositions, resting metabolic rate, and energy expenditure were also assessed. ActiGraph data indicated that subjects with NCF were more active than MCI subjects. A comparison of the IPAQ and the ActiGraph data revealed a significant correlation between these methods for total (r = 0.3315, *p* < 0.01) and moderate (r = 0.3896, *p* < 0.01) physical activity in the total population and moderate activity (r = 0.2893, *p* < 0.05) within the NCF group. No associations between these methods were found within the MCI group. Independent predictors of subjectively evaluated total physical activity were alcohol consumption (*p* = 0.0358) and socio-professional status (*p* = 0.0288), while weight (*p* = 0.0285) and the Montreal Cognitive Assessment results (*p* = 0.0309) were independent predictors of objectively measured physical activity. In conclusion, the long version of IPAQ is a more reliable tool to assess PA in subjects with NCF than those with MCI. More studies are needed to confirm this finding.

## 1. Introduction

Physical activity (PA) describes any body movement produced by the skeletal muscles that cause energy expenditure [1]. It is well known that systematic PA reduces the risk of various diseases such as diabetes, coronary heart disease, stroke, hypertension, and different types of cancer [2,3,4,5,6]. Previously, it has been shown that PA may also protect against cognitive decline and the development of dementia in older community-dwelling subjects with normal cognitive function (NCF) [7,8]. A predisposing condition to the development of dementia is a mild cognitive impairment (MCI), which refers to the cognitive impairment associated with normal age-related cognitive decline, but not severe enough to cause problems in everyday life [9]. The risk of cognitive impairment increases in middle-aged subjects and rises continuously with age [10]. It is also known that MCI is often associated with depression [11], which is a frequent finding in this population [12]. As there is no effective medical treatment for the deterioration of cognitive functions, prevention, early detection, and diagnosis of comorbidities that favour cognitive decline are essential in preventing the progression of MCI [13].

As one of the modifiable risk factors of cognitive decline, PA should be monitored using appropriate methods to assess the compliance with the recommended thresholds (150–300 min/week for moderate PA intensity or 75–150 min/week for intense PA, or an equivalent combination of both for adults 18–64 years old) [14] especially in people with MCI. The time spent in different types of PA can be assessed by subjective (questionnaires, diaries) and objective methods (motion sensors, heart rate monitors). Due to their practicality and low cost, questionnaires are used for measuring PA in epidemiological research [15], but most of them only measure a certain type of PA, e.g., in leisure time or the workplace, and only a few of them assess activity in different circumstances of everyday life, severely limiting their use [16,17]. The International Physical Activity Questionnaire (IPAQ) is one of the most widely used tools worldwide. It is a standardised questionnaire developed to collect comparable and valid PA measures within and between countries [18]. The long version of IPAQ allows data collection regarding different PA types that are part of everyday life, with questions related to activities performed at work, home, and in its surroundings, moving from place to place, in free time devoted to recreation and exercise, and time spent sitting [19], allowing the assessment of PA in subjects aged 15–69 years in the past seven days [20]. However, questionnaires cannot account for subjective over- or underestimation of one’s PA.

Daily PA can be assessed using different types of trackers, such as pedometers, smartwatch, bands, or accelerometers. The advantages of monitors used for measuring PA are, depending or their specifications, their ability to provide precise and objective information about the duration, frequency, and intensity of exercises. Hence, there is less risk of errors associated with the inaccuracy of subjective assessment [21]. Obtaining the most accurate results is possible using approved, certified medical devices, such as ActiGraph accelerometers. These accelerometers provide uninterrupted raw data on PA, using validated data processing algorithms. However, accelerometers differ in the sampling frequency, signal filtering, or wearing site. Furthermore, wrist-worn devices tend to overestimate average activity compared to hip-worn devices [22,23], and their comparatively high cost may prevent use in a larger population [24].

The estimation of PA level may vary significantly depending on the measurement methods. A systematic review comparing the objective and subjective PA measurements revealed that directly measured PA levels were either higher (40% of the studies) or lower (60% of the studies) than self-reported measures of PA, with the results varying depending on the measurement device and women showed a greater tendency to overestimate their activity [21]. Subjective and objective methods of measuring PA have been evaluated in different age groups [25,26,27] and medical conditions [28,29,30,31], indicating that different recommendations of PA are needed depending on the studied population. However, despite the positive effect of PA on cognitive decline inhibition, there is a lack of studies comparing easily accessible and cost-effective self-reported PA estimations with direct and objective methods of measuring PA in middle-aged subjects with NCF and with MCI.

Therefore, this study aimed to compare subjective (IPAQ) and objective (ActiGraph) methods of measuring total, vigorous, and moderate physical activity as well as sedentary behaviour and activity kilocalories in non-depressed middle-aged participants with and without mild cognitive impairments. A secondary goal of the study was to find factors influencing physical activity measured by the ActiGraph and the IPAQ in both groups.

## 2. Material and Methods

### 2.1. Study Population

Study participants were recruited from a group of free-living subjects of the Poznań population. Volunteers responded to advertisements distributed in local hospitals, companies, and offices. Inclusion criteria comprised: age 50–65 years, NCF (26–30 points in the Montreal Cognitive Assessment (MoCA)) or MCI (19–25 points in MoCA) [32]. Exclusion criteria included a high level of subjectively declared physical activity prior to the study (more than 10,000 steps/day or 150–300 min/week for moderate PA intensity or 75–150 min/week for intense aerobic PA, or an equivalent combination), depression (>13 points in the Hamilton Depression Rating Scale (HAM-D)), dementia, Alzheimer’s disease, Parkinson’s disease, mental illness, stroke, brain injury, kidney and liver diseases, diabetes > 10 years, decompensated hypothyroidism, anaemia, diseases that limit physical exertion, living in a nursing home, alcohol abuse, and the use of drugs affecting cognition. Initially, 101 subjects expressed an interest to participate, of which six were excluded (two subjects were older than 65 years, three had a previous stroke, and one had a history of malignant disease), ten subjects resigned, and ten were not able to start the intervention within the required time limit. Ultimately, 75 subjects (48 with NCF and 27 with MCI) provided written informed consent to participate and completed the study.

### 2.2. Ethical Issue

The study protocol was approved by the Poznan University of Medical Sciences Bioethical Committee (refs. 453/19, 882/19, 1059/19, 1167/19, 481/20, 720/20, and 752/20) and conducted according to the guidelines of the Declaration of Helsinki. Study performance and publication are in accordance with the “Strengthening the Reporting of Observational Studies in Epidemiology (STROBE)” guidelines [33].

### 2.3. Procedure

All volunteers were contacted by telephone, and those interested in participating in the study were scheduled for an appointment with a physician. The qualifying visit included a medical examination with an interview regarding past diseases, as well as an assessment of cognitive functions and the occurrence of depression. All participants were informed about the aim of the study as well as the possibility of withdrawing from the study without giving any reason at any time. Qualified participants received ActiGraph accelerometers for seven consecutive days and a self-administrated long version of the IPAQ, which was required to be completed as soon as the observation period finished to evaluate activity during the same period as measured with accelerometers. Participants completed the IPAQ themselves and delivered them to the follow-up visit, during which they could ask questions related to the questionnaire, and the investigator verified the correctness of completing the IPAQ. Moreover, all participants completed a seven-day diary of PA, noting the time when they took the device off (e.g., to take a bath) and sleep. The notes served as a reference point for accelerometer data. Participants were also instructed to follow their regular PA and not to change their habits during the observation.

### 2.4. Subjective PA Measurement

PA was assessed subjectively using the self-administrated long IPAQ. This questionnaire relates to PA performed for at least ten minutes and consists of 27 questions divided into five independent parts that assess activities related to (1) work, (2) mobility, (3) housework, house maintenance, and caring for family, (4) recreation and sports, and (5) time spent sitting. According to the guidelines for the long version of the IPAQ, each type of PA was expressed in a metabolic equivalent task [MET—minutes/week] by multiplying the coefficient assigned to each activity by the number of days it was performed per week and the duration in minutes per day [34]. One MET is equivalent to 3.5 mL O_2_ per kg body weight multiplied by minutes (ml/kg/minute of oxygen consumption (VO_2_)) and represents the energy expended while sitting at rest [35]. The total PA MET—minutes/day was calculated by summing total walking, moderate, and vigorous PA and divided by seven days, whereas activity kilocalories [kcal/day] were calculated according to the following equation: one MET = one kcal/kg body mass/hour [36]. Total and moderate PA, as well as time spent sitting, was shown in minutes/day. The methods used to evaluate the long IPAQ are detailed on the IPAQ website (www.ipaq.ki.se, accessed on 15 February 2021) [18].

### 2.5. Objective PA Measurement

The objective measurement of PA was performed using the ActiGraph GT9X Link (ActiGraph, Pensacola, FL, USA), which included a validated three-axis accelerometer and data filtering technology. Devices were initialised using a 60-second epoch of movement record, and participants were required to wear them tightly fitting on the wrist of the non-dominant hand for seven consecutive days (during day and night), except for bathing and swimming. PA recorded by the ActiGraph was expressed as the sum of counts in Freedson bouts in the registered time [counts/minute] and activity kilocalories were recorded. Periods in which the number of counts was higher than 5725 per minute were classified as high-intensity activities, between 1952 and 5724 as moderate activity, between 100 and 1951 as light activity, while less than 100 counts/minute were defined as sedentary [37]. Activity kilocalories [kcal/day] were evaluated based on the Freedson Combination 1998 algorithm, which uses the Freedson 1998 equation to calculate energy expenditure above 1952 counts per minute and the Work-Energy Theorem (kcal/minute = counts per minute × 0.0000191 × weight) for counts per minute below 1951. Sleep time was excluded from the analysis of PA. The processing and evaluation of the collected data were performed in the ActiLife 6.13.4 software (ActiGraph, Pensacola, FL, USA) using independently developed and proven Freedson 1998 algorithms [37].

### 2.6. Anthropometric Parameters and Body Composition

Basic anthropometric parameters (such as body weight and body height) were measured and the body mass index (BMI) was calculated by dividing the weight by the height in meters squared. Body composition (fat mass (FM) and fat-free mass (FFM)) was evaluated by the air displacement plethysmography method using the BOD POD analyser (Cosmed, Albano Laziale, Italy), and body density, percentage of body fat (% FM), and fat-free mass (% FFM) were calculated.

### 2.7. Resting Metabolic Rate

The indirect calorimetry method was used to measure the resting metabolic rate (RMR) using the QUARK RMR analyser (Cosmed, Albano Laziale, Italy). This method provided accurate results in a non-invasive way, through the measurement of oxygen consumption and carbon dioxide production. The device has been calibrated using a certified calibration syringe according to the manufacturer′s recommendations. RMR was monitored instantaneously on spontaneously breathing participants with a flow-dilution canopy hood. The exhaled air was transported to the device, where the flow rate was measured with a digital turbine flowmeter. Before the examination, participants were not allowed to perform PA and consume meals for at least 12 h. During the test, they were asked to lay comfortably on their back and without moving for a minimum of ten minutes.

### 2.8. Evaluation of Cognition and Depression

The cognition in all participants was assessed using MoCA, which evaluates eight different cognitive areas and takes approximately ten minutes to complete. The maximum score of MoCA is 30 points, a score of 26 or more indicates NCF, and a score of 19–25 points indicates MCI. The cut-off point allows the detection of MCI with a sensitivity of 80.48% and a specificity of 81.19% [38].

HAM-D was used to select subjects with symptoms of depression. It includes 17 questions, and the answers are scored on a scale from 0 to 4, with a score below seven points suggesting no disorders, while higher scores indicate depression of varying severity (7–12 points: mild depression, 13–17 points: moderate depression, 18–29 points: severe depression, 30–52 points: very severe depression). Both tests were performed by a qualified researcher.

### 2.9. The Minimum Sample Size

Assuming α = 0.05, β = 0.2, the difference of predicted means equals 25%, and the expected value of standard deviation (SD) equals 30% of the mean, 23 subjects should be recruited in each study group to obtain a power of 80%. Considering a 20% drop-out rate, a total of 29 subjects per group should be included in the study. Calculations were performed using the G*Power software (version 3.1.9.2, University of Kiel, Kiel, Germany). Predicted mean and expected standard deviation were calculated based on the publication by Hagstromer et al. [39] and our small pilot study).

### 2.10. Statistical Analysis

All statistical analyses were performed using Statistica 13.0 (TIBCO Software Inc., Palo Alto, CA, USA) and PQStat (PQStat Software, Poznań, Poland) softwares, with the two-tailed level of significance set at *p* < 0.05. Data are presented as mean and SD as well as a median and interquartile range (Q1–Q3). The Shapiro–Wilk test was performed to verify the normality of the distribution of the variables, and as most data were non-normally distributed, non-parametric statistical tests were used. MCI and NCF participants were compared using the Mann–Whitney U test. In addition, Cohen’s d statistic was calculated to assess the effect size of the difference between the MCI and NCF groups.

A Cohen d value of one indicates that the two groups differ by one standard deviation, d = 0.2 indicates small size effect, d = 0.5 represents medium, and d = 0.8 indicates large effect size. Positive results indicate higher and negative lower mean results in the MCI group than in the NCF group.

Both PA measuring methods were compared in terms of assessing moderate and total physical activity as well as sedentary behaviour and activity kilocalories. The comparison was made for the total population as well as separately in NCF and MCI groups. As none of the participants achieved vigorous PA in the ActiGraph, it was not possible to compare the two methods in the assessment of this type of effort. Moreover, IPAQ assesses only sedentary behaviour and moderate and vigorous activity; therefore, no comparisons of light activity were performed.

Bland–Altman plots were used to examine the agreement between the compared methods of measuring PA levels in the NCF and MCI participants [40]. Bland–Altman plots compared daily time spent in sedentary behaviour and moderate PA as well as activity kilocalories evaluated by the IPAQ and assessed by the ActiGraph, plotting the differences between the measures (in the same metric) against the average of the measures. To describe the total error between the two methods, 95% limits of agreement were calculated [39,41]. Correlations between total and moderate PA as well as sedentary behaviour and activity kilocalories assessed by the accelerometer and the IPAQ were measured using Spearman′s rank correlation test. Moreover, Cohen’s kappa coefficient was calculated for comparison of tertiles of data from each physical activity category to measure interrater reliability between the accelerometer and IPAQ in the classification of PA categories and estimation of activity kilocalories. Finally, to show concordance between the classification of the IPAQ and the ActiGraph, data of tertiles based on the distribution of the data from these two instruments regarding moderate and total physical activity, sedentary behaviour and activity kilocalories were compared using Kendall’s tau-b.

Univariate linear regression analysis for the total population as well as NCF and MCI groups was performed to evaluate the relationship of selected variables (sex, age, weight, place of living, family situation, education, socio-professional status, alcoholic drinks, smoking, HAM-D points, MoCA points, RMR, and EE) with total physical activity measured by the IPAQ or total counts in Freedson bouts per minute measured by the ActiGraph. Then, variables from the univariate analysis with *p* < 0.1 were entered into multivariate linear regression. Moreover, correlations between total physical activity measured by the IPAQ, as well as by the ActiGraph, and selected variables were checked using Spearman′s test in the total population, MCI, and NCF groups.

## 3. Results

### 3.1. Characteristics of the Study Population

The baseline characteristics of the study population (*n* = 75) are presented in Table 1 and Table 2. Of the 75 participants, there were 48 women and 27 men with a mean age of 58 ± 5 years. Most participants had NCF (64%), while MCI occurred in 27 (36%) participants. Moreover, 28 participants had normal BMI, 30 were classified as overweight, and 17 as obese, while the mean BMI was 26.96 ± 5.47 kg/m^2^. As presented in Table 2, most participants came from a large city, were in a relationship, had higher education, were professionally active, and consumed small amounts of alcohol, but only a few of them smoked. Besides, according to the ActiGraph measurements, all participants achieved the recommended levels of PA, while according to the IPAQ, 90% of participants achieved the PA thresholds (150–300 min/week for moderate PA intensity or 75–150 min/week for intense PA, or an equivalent combination of both) [14]. Despite the declaration of low before the study, 85% of participants achieved an average number of daily steps above the recommended 10,000 steps per day.

### 3.2. Comparison of MCI and NCF Participants

There were no differences between groups in most of the analysed parameters, except age, sex, MoCA points, and education. Table 3 presents the comparison of PA assessed by the IPAQ between NCF and MCI groups. There were no differences between these two groups for any of the evaluated parameters (*p* > 0.05). Table 4 shows the comparison of PA evaluated by the ActiGraph between NCF and MCI groups. MET rate and total physical activity were significantly higher in NCF participants than in the MCI group (1.73 ± 0.16 vs. 1.56 ± 0.13, *p* < 0.0001 and 586 ± 291 vs. 351 ± 233 counts/minute, *p* = 0.0003, respectively). Time spent in moderate activity was longer in the NCF group comparing to the MCI group (188 ± 68 vs. 128 ± 52 min/day, *p* = 0.0003 and 19.3 ± 6.7 vs. 13.2 ± 5.2%, *p* = 0.0001, respectively), while sedentary time was shorter in NCF participants than in the MCI group (246 ± 86 vs. 310 ± 78 min/day, *p* = 0.0004 and 25.0 ± 7.1 vs. 32.1 ± 7.4%, *p* = 0.0001). Moreover, participants in the MCI group had a significantly lower average number of steps than the NCF group (12,358 ± 2963 vs. 14,423 ± 3404, *p* = 0.0079).

### 3.3. Comparison of Subjective and Objective Methods of Measuring Physical Activity

**Figure 1:**
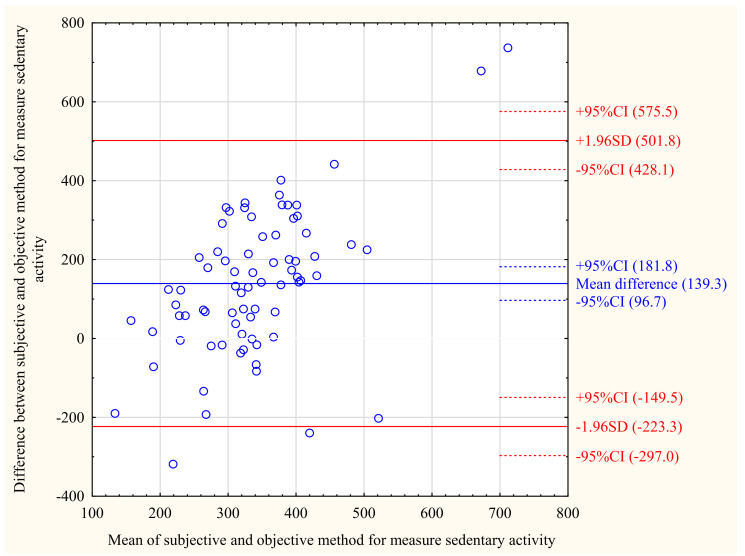
Bland–Altman plot for sedentary behaviour [min/day] measured by the IPAQ and ActiGraph in total study population (*n* = 75).

**Figure 2:**
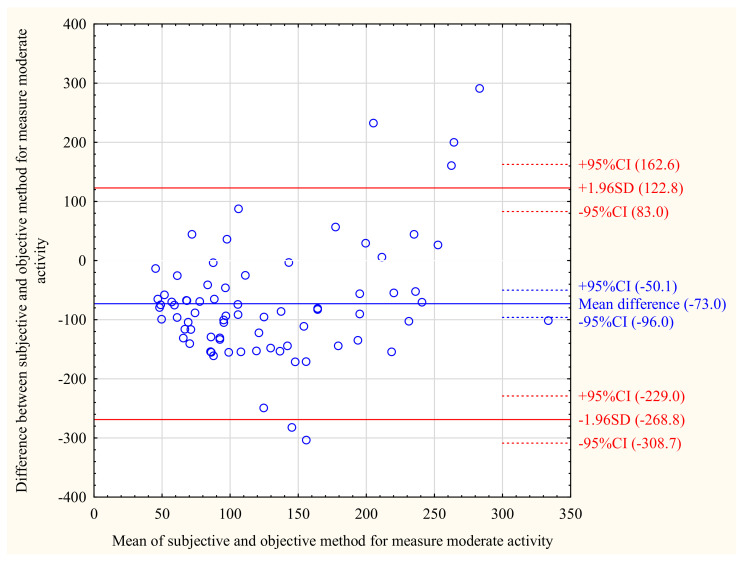
Bland–Altman plot for moderate activity [min/day] measured by the IPAQ and ActiGraph in total study population (*n* = 75).

**Figure 3:**
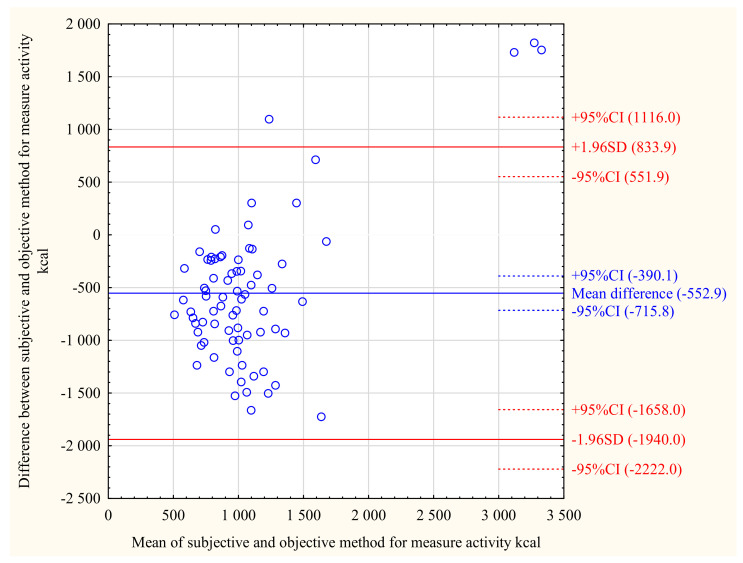
Bland–Altman plot for activity kilocalories per day measured by the IPAQ and ActiGraph in total study population (*n* = 75).

Figure 1, Figure 2 and Figure 3 show the Bland–Altman plot for sedentary behaviour, moderate PA, and activity kilocalories measured by the ActiGraph and the IPAQ in the total population. The same analyses for MCI and NCF groups are shown in the Supplementary Materials (Figures S1–S6). The Bland–Altman plots for the total population show that moderate activity (mean difference = −73.03 min/day) and activity kilocalories (mean difference = −552.9 kcal/day) were lower, while sedentary behaviour (mean difference = 139.3 min/day) was higher when measured by the IPAQ than by the ActiGraph. As shown in the Supplementary Materials (Figure S1–S6), this trend was similar in the MCI and NCF groups; however, in the MCI group, the mean difference values were closer to the absolute agreement level than in the NCF group. The individual differences between the objective and subjective measurements were within acceptable limits for most participants for activity categories as well as for activity kilocalories.

Table 5, Table 6 and Table 7 show a comparison between the objective and subjective methods of measuring total and moderate PA as well as sedentary behaviour and activity kilocalories. Significant correlations between results obtained by the IPAQ and the ActiGraph were observed for total (r = 0.3315, *p* < 0.01) and moderate (r = 0.3896, *p* < 0.01) PA in the total population and moderate activity (r = 0.2893, *p* < 0.05) in the NCF group (Table 5). Table 6 shows the kappa (κ) coefficients between the tertiles of the objective and subjective method for the measurement of PA. A significant agreement between these two methods was observed for total (Cohen′s κ = 0.32, *p* < 0.01) and moderate (Cohen′s κ = 0.41, *p* < 0.001) PA in the total population, while no agreement was seen for any of compared PA categories in the MCI group (Table S1). There was also agreement between these methods for moderate activity in the NCF group (Table S2). As shown in Table 7, there was a statistically significant relationship between subjective and objective measurement of PA for total (Kendall’s tau-b = 0.2897, *p* < 0.001) and moderate (Kendall’s tau-b = 0.3581, *p* < 0.0001) PA in the study population, but it was not seen in MCI group (Table S3). A relationship between these methods was also observed for total (Kendall’s tau-b = 0.2269, *p* < 0.05) and moderate (Kendall’s tau-b = 0.3183, *p* < 0.01) PA in the NCF group (Table S4).

### 3.4. Relationship between Total Physical Activity and Selected Variables

Univariate and multivariate linear regression analyses assessing the relationship of selected variables with total PA measured by the IPAQ [MET—minute/day] or by the ActiGraph [counts/minute] for the total population, are presented in Table 8, Table 9, Table 10 and Table 11.

Variables with a *p*-value < 0.1 in the univariate analysis were included in the multivariate regression analysis. In the total population, alcohol consumption (*p* = 0.0358) and socio-professional status (*p* = 0.0288) appeared to be independent predictors of total PA measured by the IPAQ (Table 9), while weight (*p* = 0.0285) and MoCA points (*p* = 0.0309) were identified as independent predictors of total physical activity measured by the ActiGraph (Table 11). There was no relationship between RMR results and either subjectively or objectively measured total PA in the total study population. As shown in the Supplementary Materials (Tables S5–S10), RMR (*p* = 0.02490) was an independent predictor of total PA measured by the ActiGraph in the MCI group, while weight (*p* = 0.0036), socio-professional status (*p* = 0.0104) and HAM-D points (*p* = 0.0077) in the NCF group. There were no independent predictors for subjectively evaluated PA in both groups.

There were statistically significant correlations between total physical activity measured by the ActiGraph and weight (r = -0.5244, *p* < 0.0001), BMI (r = −0.4393, *p* < 0.0001), FM [kg] (r = −0.3928, *p* = 0.0005), FFM [kg] (r = −0.4173, *p* = 0.0002), MoCA points (r = 0.3303, *p* = 0.0038), and RMR (r = −0.4844, *p* < 0.0001) in the total population. Correlations between objectively measured PA and weight (r = −0.5634, *p* < 0.0001), BMI (r = −0.5200, *p* = 0.0002), FM [kg] (r = −0.4620, *p* = 0.0009), FFM [kg] (r = −0.5037, *p* = 0.0003), HAM-D points (r = 0.4386, *p* = 0.0018), as well as RMR (r = −0.4591, *p* = 0.0010) were also seen in the NCF group, while weight (r = −0.4709, *p* = 0.01316), FFM [kg] (r = −0.4159, *p* = 0.0310), alcohol consumption (r = −0.3902, *p* = 0.0442), and RMR (r = −0.5513, *p* = 0.0029) were seen in the MCI group. Moreover, no correlation between total PA measured by the IPAQ and any of the measured parameters was observed in the total population as well as in the NCF and the MCI groups.

### 3.5. Relationship between MoCA Points and Selected Variables

Univariate linear regression analysis assessing the relationship of selected variables with MoCA points was performed, and variables with *p*-value < 0.1 were included in the multivariate regression analysis. Age (*p* = 0.0038), education (*p* = 0.0007), and total PA measured by the ActiGraph (*p* = 0.0350) appeared to be independent predictors of MoCA points in the total population (Table 12 and Table 13). There were no independent predictors for MoCA points in the MCI and the NCF groups (Tables S11–S14).

There were statistically significant correlations between MoCA points and age (r = −0.4272, *p* = 0.0001) and total PA measured by the ActiGraph (r = 0.3303, *p* = 0.0038) in the total population. Correlations between MoCA points and FM [%] (r = −0.6350, *p* = 0.0004), FFM [%] (r = 0.6350, *p* = 0.0004), FFM [kg] (r = 0.7462, *p* < 0.0001), as well as RMR (r = 0.4653, *p* = 0.0145) were also seen in the MCI group, while no correlation was observed in the NCF group.

## 4. Discussion

In this study conducted on middle-aged subjects with NCF or MCI, PA categories obtained in the IPAQ (subjective method) were compared with the ActiGraph (objective method) results, indicating a significant but low correlation between the measurement methods for total and moderate PA in the total population and total PA in the NCF group. No statistically significant association between these two methods was observed for any of the PA categories compared in the MCI group. Moreover, moderate activity and activity kilocalories were lower, while sedentary behaviour was higher when measured by the IPAQ than by the ActiGraph. Besides, according to the ActiGraph results, participants with NCF were more active and spent less time sedentary compared to participants with MCI, while IPAQ results did not show any differences in PA categories between groups.

A high level of PA can protect from cognitive decline. A recent meta-analysis indicated that PA is effective in improving cognitive function in adults aged > 50 years, regardless of cognitive performance [42]. In another meta-analysis of 15 prospective studies, the authors found a 38% risk reduction in cognitive decline in participants who performed a high level of PA compared to sedentary participants [43]. However, the comparative data on different categories of PA recording in participants with NCF and MCI are scarce. In a recent study of 49 subjects with MCI or dementia, van der Wardt et al. [44] reported moderate and strong correlations between the accelerometer and IPAQ. This is in contrast to our results, but we compared PA categories, while van der Wardt et al. [44] only compared METs rate estimated in the IPAQ with steps amount measured by accelerometer. Moreover, physical activity in our study was assessed by a different type of accelerometer, and another type of questionnaire was used to assess participants’ cognition including only participants with MCI, without dementia.

Previous studies indicated that the directly measured levels of PA were both lower [39,45] and higher [46] than self-reported estimations, which creates the problem of selecting the appropriate measurement method. In a systematic review, Prince et al. [21] found that the self-report measures of PA more often were higher than directly measured PA levels. Results varied depending on the device used, and the differences in woman’s estimations of PA in relation to the objective measurements were greater than in men. According to another systematic review, the correlations between the results estimated with questionnaires and obtained with accelerometers were stronger in men compared to women and in younger compared to older subjects [47]. Sabia et al. [48] concluded that the associations between accelerometer-assessed and questionnaire-based PA depend on the type of measured PA, and the results are more compliant for more energetic activities. Accelerometers are considered to be one of the best devices used to validate self-report questionnaires for estimation PA levels [49]. However, the comparison of subjectively estimated PA with levels measured by accelerometers has also been criticised as accelerometers and self-administrated reports assessed different aspects of PA [50]. The differences may be due to the method for calculating total PA, which for the ActiGraph data, includes sedentary time, while for the IPAQ, sitting time is not included in total PA calculations. Moreover, the results obtained from accelerometers worn on different parts of the body might differ [51]. According to Tudor-Locke et al. [52], accelerometers worn on the hips are more accurate in counting steps than those worn on wrists. Algorithms used to analyse the data obtained from accelerometers can also affect the results [50]. Since we did not find the correlation between subjectively and objectively evaluated total PA and sedentary behaviour in the MCI and the NCF subgroups, larger studies are warranted to confirm these findings.

Subjective estimation of PA can be performed using diaries or questionnaires, and one of the most commonly used and wildly validated questionnaires is the IPAQ. However, questions in IPAQ regard only the time and frequency spent in vigorous and moderate PA, which is a subjective rating of performed activity intensity. Previous research results showed that this perception of intensity may be higher for subjects with excessive body weight or low aerobic capacity [39]. It should also be noted that the involvement of a trained interviewer may result in more reliable results [34]. Since the long version of the IPAQ provides the ability to collect data on different types of PA that are part of everyday life, it can be useful for evaluating intervention trials when more details about changes in different intensities of PA are required [53].

Previously, several studies compared the results of PA measurements obtained by the ActiGraph and the IPAQ. Hagstromer et al. [39] noticed a modest but significant correlation for vigorous, moderate, and total PA between the ActiGraph and the IPAQ in the Swedish adult population under the age of 65. In our research, we found similar relationships between moderate and total PA. However, none of the participants achieved vigorous activity according to the ActiGraph. Therefore, we could not compare this PA level with the IPAQ results.

Furthermore, Skender et al. [47] in a systematic review reported that Spearman′s correlations between PA measured by the ActiGraph and the results obtained in the questionnaire among the adult population were stronger with the extension of the observation period, suggesting the need for a longer study. Our research results are not in line with Ekelund et al. [45] who reported that in 87 healthy males, aged 20 to 69 years, randomly selected from different work places, time spent in sedentary assessed by accelerometer was significantly correlated with self-administrated time spent sitting. Lipert et al. [25] found that regardless of age, weight, and type of obesity, both free-living women and men aged 45–64 had higher activity kilocalories estimated by IPAQ than measured by the accelerometer, which is not consistent with our results. Nevertheless, in that study, accelerometers were attached to the waist, near to the centre of body weight, while in our study participants wore devices on the wrists, which could be a possible reason for the lack of concordance of the obtained results.

Given that we recruited our study subjects who reported low levels of PA before enrolment in the study, it is possible that the awareness of measuring PA during the observation period increased the participants′ activity [54]. Another explanation for the weak association between our results may be that wrist-worn accelerometers overestimate the average activities compared to hip-worn devices [22]. Generally, to obtain the most complete information, Skender et al. recommended the usage of both questionnaires and accelerometers to evaluate PA levels [47].

Activity monitors enable older people to achieve PA goals by promoting self-efficacy and PA [55,56]. Indeed, in a systematic review, Cooper et al. [57] indicated that accelerometers, when used alone or in combination with other interventions, increase PA in the elderly. However, only 20% of adults over 55 reported using PA trackers [58]. Technological barriers such as the lack of a suitable device or the required web application can make it difficult for middle-aged and older subjects to use trackers [54]. Moreover, cognitive impairment in the elderly might also increase the difficulty of using a PA tracker, as well as understanding and following PA recommendations.

It is well known that MCI prevalence increases with age [59] but less with higher education [42]. Moreover, several studies showed that cognitive impairment is more common in men than in women [13,60]. However, other studies reported that the prevalence of Alzheimer’s disease is higher in women than in men [61,62]. We confirmed these relationships in our study, since participants in the MCI group were significantly older and had lower education than participants in the NCF group. There were more women in the NCF than in the MCI group. We recruited subjects aged 50–65, since in this age group, it is possible to change the trajectory of cognitive decline, while the risk of cognitive impairment is highest in subjects over 65, yet their ability to change the trajectory might be more limited [59]. Early intervention may inhibit the progression of cognitive disorders in subjects with MCI. Therefore, further intervention studies should also focus on this population.

Previous studies have shown that PA can be determined by many socio-demographic factors such as age, sex, education level, ethnicity, and depression [63,64], as well as many other predictors selected within different populations [64]. We consequently performed univariate linear regression analysis in the total population as well as NCF and MCI groups to evaluate the relationship of selected variables (sex, age, weight, place of living, family situation, education, socio-professional status, alcoholic drinks, HAM-D points, MoCA points, and RMR) with total physical activity measured by the IPAQ or by the ActiGraph. Our study indicated different predictors of physical activity for MCI and NCF participants, but also for the method used for PA assessment.

The strength of our study was the use of validated research tools for subjective and objective PA measurement [37,65]. The long version of the IPAQ is one of the most widely used questionnaires assessing PA and allows for a detailed evaluation of activity in various circumstances of everyday life [16,19], while the ActiGraph GT9X Link accelerometer is one of the newest devices and most commonly used, approved, and certified within the European Union and the United States [66]. An additional strength of our study was the detailed characterisation of the study populations and the use of confirmed, non-invasive methods for assessing energy expenditure and resting metabolic rate. Besides, we excluded subjects with depression, since it has additional effects on cognition [12,67,68] and increases the risk of MCI progression to dementia [69]. Furthermore, this is one of the first studies that compares the subjective and objective measurement of different categories of performed PA among middle-aged subjects with MCI or NCF.

There are some limitations to this study. Firstly, the study sample was relatively small for subgroup analysis, and there were more participants in the NCF than in the MCI group. However, as mentioned earlier, this is the first pilot cross-sectional study that compared ActiGraph with IPAQ in subjects with MCI or NCF. Therefore, further studies are needed to confirm our findings. Moreover, we recruited to the study more women than men. However, it is well known that women more frequently participate in research trials [70]. Furthermore, our research was only performed over a short period; however, the length of our study was comparable to other studies comparing the levels of physical activity measured with the ActiGraph and the IPAQ [25,39]. Likewise, we recruited patients in a narrow age range. Therefore, it is not possible to generalize the results for the entire population. Additionally, we did not perform control for multiple comparisons due to size limitations. Moreover, participants in both groups increased their activity levels during the study period above the pre-study level. Due to the short duration of the study, it is impossible to determine whether the measured PA was the actual daily activity of the participants. Furthermore, most participants achieved a higher step number than declared before recruitment, which could be caused by awareness of PA measuring. Moreover, the GT9X sensing technology relies on the proximity of the sensor electrode to the skin; therefore, vibrations or movements with simultaneous loose wear of the band may impair accuracy [71]. Finally, PA in our study was measured with wrist-worn accelerometers; whereas, the previous study shows that the specificity and the sensitivity values of the wrist-worn and hip-worn accelerometers were lower than those of the thigh accelerometer [72].

## 5. Conclusions

In summary, we found a relationship between IPAQ and the ActiGraph for moderate activity in the total study population and the NCF group, while no significant agreement between these two methods was observed for any of the compared PA levels in the MCI group. This may indicate that the long version of IPAQ is a more reliable tool to assess PA in subjects with NCF than those with MCI. The present study is the first investigation examining the reliability of IPAQ and the AcitGraph for the assessment of total PA and particular categories of PA in adults with NCF and MCI. Longer and larger studies should be performed to further support the present findings.

## Figures and Tables

**Table 1:** Baseline characteristics of the study population.

	Total (*n* = 75)		MCI (*n* = 27)		NCF (*n* = 48)		*p* *	Effect ^1^ Size
	Mean ± SD	Median (Q1–Q3)	Mean ± SD	Median (Q1–Q3)	Mean ± SD	Median (Q1–Q3)		
Age [years]	58 ± 5	57 (53–62)	60 ± 4	60 (56–63)	56 ± 5	56 (52–61)	0.0018	0.9
Weight [kg]	77.4 ± 18.4	78.4 (61.4–84.8)	78.3 ± 17.2	79.8 (64.8–89.3)	76.9 ± 19.3	74.3 (60.9–84.8)	0.3861	0.1
Height [cm]	169 ± 10	168 (163–176)	169 ± 11	169 (156–178)	169 ± 9	168 (164–175)	0.8553	0.0
BMI [kg/m^2^]	26.96 ± 5.47	25.87 (23.00–28.21)	27.34 ± 5.75	26.00 (25.18–32.08)	26.75 ± 5.35	25.73 (23.00–28.21)	0.6310	0.2
Fat mass [%]	35.1 ± 10.5	35.0 (26.3–44.2)	35.6 ± 11.7	31.3 (26.3–45.1)	34.9 ± 9.9	35.0 (25.6–42.4)	0.9604	0.1
Fat free mass [%]	64.9 ± 10.5	65.0 (55.8–73.7)	64.4 ± 11.7	68.7 (54.9–73.7)	65.2 ± 9.9	65.0 (57.6–74.5)	0.9604	−0.1
Fat mass [kg]	27.9 ± 12.6	24.7 (17.7–37.1)	28.6 ± 13.7	24.3 (17.9–37.1)	27.5 ± 12.2	24.8 (16.8–37.3)	0.8815	0.1
Fat free mass [kg]	49.5 ± 11.7	46.8 (40.9–59.0)	49.7 ± 11.5	49.9 (40.9–61.4)	49.4 ± 11.9	45.7 (41.2–53.9)	0.7363	0.0
RMR [kcal/d]	1665 ± 304	1675 (1410–1883)	1685 ± 279	1785 (1412–1883)	1653 ± 320	1651 (1408–1857)	0.4300	0.1
MoCA [points]	26 ± 3	27 (24–29)	23 ± 2	24 (22–24)	28 ± 1	29 (27–29)	<0.0001	−3.2
HAM–D [points]	5 ± 3	4 (2 –7)	5 ± 3	4 (3–6)	4 ± 3	4 (2–7)	0.1765	0.3

BMI—body mass index; EE—energy expenditure; HAM–D—Hamilton depression rating scale; MCI—mild cognitive impairment; MoCA—Montreal Cognitive Assessment; NCF—normal cognitive function; Q1–Q3—interquartile range; RMR—resting metabolic rate; SD—standard deviation. * *p* for baseline differences between subjects with MCI and NCF in the Mann–Whitney U test. ^1^ Cohen d test.

**Table 2:** Socio-economic characteristics of the study population.

	Total (*n* = 75)	MCI (*n* = 27)	NCF (*n* = 48)	*p* *
	*n* (%)			
Sex [% of women]	48 (64.0)	13 (48.2)	35 (72.9)	0.0320
Place of residence				
City > 500,000 inhabitants	47 (62.6)	12 (44.4)	35 (72.9)	0.0982
City 50,000–500,000 inhabitants	3 (4.0)	2 (7.4)	1 (2.1)	
Town < 50,000 inhabitants	14 (18.7)	7 (25.8)	7 (14.6)	
Village	11 (14.7)	6 (22.2)	5 (10.4)	
Family status				
Single	18 (24.0)	5 (18.5)	13 (27.1)	0.1758
Married	53 (70.7)	22 (81.5)	31 (64.6)	
Informal relationship	3 (5.3)	0 (0.0)	4 (8.3)	
Education				
Higher	56 (74.6)	14 (51.9)	42 (87.5)	0.0019
Secondary	17 (22.7)	11 (40.7)	6 (12.5)	
Primary	2 (2.7)	2 (7.4)	0 (0.0)	
Social and professional status				
Active	52 (69.3)	18 (66.7)	34 (70.8)	0.1115
Pensioner	18 (24.0)	9 (33.3)	9 (18.8)	
Unemployed	5 (6.7)	0 (0.0)	5 (10.4)	
Smoking	4 (5.3)	2 (7.4)	2 (4.2)	0.5488
Alcohol consumption ^1^	40 (53.3)	12 (44.4)	28 (58.3)	0.2471

MCI—mild cognitive impairment; NCF—normal cognitive function. * *p* for baseline differences between subjects with MCI and NCF in the Chi-square test. ^1^ Cohen d = 0.0.

**Table 3:** Physical activity results assessed by the IPAQ in the study population.

	Total (*n* = 75)		MCI (*n* = 27)		NCF (*n* = 48)		*p* *	Effect Size ^1^
	Mean ± SD	Median (Q1–Q3)	Mean ± SD	Median (Q1–Q3)	Mean ± SD	Median (Q1–Q3)		
Moderate activity [MET—min/day]	340 ± 357	215 (83–456)	344 ± 343	248 (103–369)	338 ± 368	193 (79–466)	0.6815	0.0
Moderate activity [min/day]	93 ± 97	60 (21–141)	92 ± 95	63 (23–99)	94 ± 99	58 (21–146)	0.7825	0.0
Vigorous activity [MET—min/day]	115 ± 205	57 (0–137)	78 ± 113	6 (0–137)	137 ± 241	69 (0–154)	0.3367	−0.3
Vigorous activity [min/day]	14 ± 25	6 (0–17)	10 ± 14	1 (0–17)	17 ± 30	8 (0–19)	0.3864	−0.3
Sedentary behaviour [min/day]	409 ± 167	394 (300–490)	369 ± 133	369 (283–480)	431 ± 181	420 (304–516)	0.1814	−0.4
Total physical activity [MET—min/day]	619 ± 496	481 (280–750)	563 ± 451	444 (229–733)	651 ± 522	486 (353–770)	0.3341	−0.2
Total physical activity [min/day]	163 ± 126	124 (75–214)	149 ± 131	113 (69–197)	170 ± 123	135 (79–223)	0.2943	−0.2
Total physical activity [kcal/day]	798 ± 779	621 (426–848)	690 ± 451	621 (313–956)	859 ± 912	614 (443–842)	0.6789	−0.2

IPAQ–International Physical Activity Questionnaire; MCI—mild cognitive impairment; MET—metabolic equivalent of task; NCF—normal cognitive function; Q1–Q3—interquartile range; SD—standard deviation.* *p* for differences between subjects with MCI and NCF in the Mann–Whitney U test. ^1^ Cohen d test.

**Table 4:** Physical activity results assessed by the ActiGraph in the study population.

	Total (*n* = 75)		MCI (*n* = 27)		NCF (*n* = 48)		*p* *	Effect Size ^1^
	Mean ± SD	Median (Q1–Q3)	Mean ± SD	Median (Q1–Q3)	Mean ± SD	Median (Q1–Q3)		
Wear [%]	96.8 ± 6.0	98.9 (96.5–99.6)	97.0 ± 4.8	98.8 (96.9–99.7)	96.7 ± 6.7	99.0 (96.5–99.6)	0.8680	0.2
Kcal/day	1351 ± 403	1257 (1041–1581)	1207 ± 349	1150 (911–1460)	1432 ± 412	1335 (1133–1712)	0.0247	−0.6
MET rate	1.67 ± 1.68	1.65 (1.53–1.77)	1.56 ± 0.13	1.55 (1.45–1.68)	1.73 ± 0.16	1.71 (1.61–1.85)	<0.0001	−1.2
Total physical activity [counts/min]	501 ± 293	444 (273–720)	351 ± 233	291 (165–532)	586 ± 291	547 (93–765)	0.0003	−0.8
Sedentary behaviour [min/day]	269 ± 88	264 (208–333)	310 ± 78	309 (277–351)	246 ± 86	232 (187–287)	0.0004	0.8
Light activity [min/day]	536 ± 72	528 (499–594)	529 ± 83	519 (499–566)	540 ± 65	535 (499–596)	0.4730	−0.1
Moderate activity [min/day]	166 ± 69	158 (112–213)	128 ± 52	124 (86–173)	188 ± 68	179 (140–241)	0.0003	−1.0
Sedentary behaviour [%]	27.6 ± 8.0	26.8 (21.7–33.7)	32.1 ± 7.4	33.0 (28.5–36.8	25.0 ± 7.1	24.3 (19.6–28.7	0.0001	1.0
Light activity [%]	55.3 ± 5.3	55.7 (51.3–59.0)	54.7 ± 5.5	54.3 (52.0–57.1)	55.7 ± 5.2	56.1 (51.2–59.7	0.6509	−0.2
Moderate activity [%]	17.1 ± 6.8	15.7 (11.4–22.8)	13.2 ± 5.2	11.8 (8.6–15.7)	19.3 ± 6.7	19.2 (14.3–23.7)	0.0001	−1.1
Steps/d	13,680 ± 3382	13,592 (10,723–16,764)	12,358 ± 2963	11,795 (9983–15,076)	14,423 ± 3404	14,242 (12,064–17,175)	0.0079	−0.6

MET—metabolic equivalent of task; MCI—mild cognitive impairment; NCF—normal cognitive function; Q1–Q3—interquartile range; SD—standard deviation.* *p* for differences between subjects with MCI and NCF in the Mann–Whitney U test. ^1^ Cohen d test.

**Table 5:** Spearman’s rank correlation between the objective and subjective methods for measure physical activity in the study population.

	Total (*n* = 75)		MCI (*n* = 27)		NCF (*n* = 48)	
	r	*p*	r	*p*	r	*p*
Total physical activity ^1^	0.2893	0.0118	0.1972	0.3242	0.2601	0.0742
Sedentary behaviour ^2^	0.0095	0.9357	0.0177	0.9301	0.0986	0.5050
Moderate activity ^3^	0.3315	0.0037	0.2053	0.3044	0.3896	0.0062
Kcal/day ^4^	0.0704	0.5486	−0.0250	0.9014	0.1397	0.3436

MCI—mild cognitive impairment; NCF—normal cognitive function; ^1^ total physical activity [counts/minute] measured by the ActiGraph vs. total physical activity [MET min/day] measured by IPAQ, ^2^ sedentary behaviour [min/day] measured by the ActiGraph vs. IPAQ, ^3^ moderate activity [min/day] measured by the ActiGraph vs. IPAQ, ^4^ activity kilocalories per day measured by the ActiGraph vs. IPAQ.

**Table 6:** Kappa (κ) coefficients between the tertiles of the objective and subjective method for measure physical activity in the total study population.

	κ (95% CI)	SE	Z	*p*
Total physical activity ^1^	0.32 (−0.11–0.53)	0.12	2.80	0.0051
Sedentary behaviour ^2^	−0.04 (−0.27–0.18)	0.12	−0.38	0.7063
Moderate activity ^3^	0.41 (0.22–0.60)	0.12	3.53	0.0004
Kcal/day ^4^	0.10 (−0.12–0.32)	0.12	0.87	0.3865

^1^ Total physical activity [counts/minute] measured by the ActiGraph vs. total physical activity [MET min/day] measured by IPAQ, ^2^ sedentary behaviour [min/day] measured by the ActiGraph vs. IPAQ, ^3^ moderate activity [min/day] measured by the ActiGraph vs. IPAQ, ^4^ activity kilocalories per day measured by the ActiGraph vs. IPAQ.

**Table 7:** Kendall’s tau-b coefficients between the tertiles objective and subjective method for measuring physical activity in the study population.

	Kendall’s Tau-B	*p*
Total physical activity ^1^	0.2897	0.0002
Sedentary behaviour ^2^	−0.0386	0.6239
Moderate activity ^3^	0.3581	<0.0001
Kcal/day ^4^	0.0880	0.2640

^1^ Total physical activity [counts/minute] measured by the ActiGraph vs. total physical activity [MET min/day] measured by IPAQ, ^2^ sedentary behaviour [min/day] measured by the ActiGraph vs. IPAQ, ^3^ moderate activity [min/day] measured by the ActiGraph vs. IPAQ, ^4^ activity kilocalories per day measured by the ActiGraph vs. IPAQ.

**Table 8:** Univariate linear regression analysis assessing the relationship between total physical activity [MET—min/day] measured by IPAQ and selected variables in the total study population (*n* = 75).

	β	SE	t	*p*
Sex ^1^	−0.1178	0.1162	−1.0133	0.3143
Age [years]	0.1110	0.1163	0.9539	0.3433
Weight [kg]	0.0160	0.1170	0.1367	0.8916
Place of living ^2^	−0.0142	0.1170	−0.1214	0.9037
Family situation ^3^	0.0828	0.1166	0.7100	0.4800
Education ^4^	−0.1034	0.1164	−0.8882	0.3774
Socio-professional status ^5^	−0.2201	0.1142	−1.9275	0.0578
Alcoholic drinks [units/week]	0.2084	0.1145	1.8202	0.0728
HAM–D [points]	0.0844	0.1166	0.7237	0.4716
MoCA [points]	−0.0781	0.1167	−0.6695	0.5053
RMR [kcal/d]	0.0761	0.1167	0.6517	0.5166

HAM–D—Hamilton depression rating scale; IPAQ—International Physical Activity Questionnaire; MoCA–Montreal Cognitive Assessment; RMR—resting metabolic rate; SE—standard error. ^1^ Men vs. women, ^2^ city vs. village, ^3^ in a relationship vs. single, ^4^ higher education vs. secondary + primary education, ^5^ employed vs. unemployed.

**Table 9:** Multivariate linear regression analysis assessing the relationship between total physical activity [MET—min/day] measured by IPAQ and selected variables in the total study population (*n* = 75).

	β	SE	t	*p*
Alcoholic drinks [units/week]	0.2404	0.1124	2.1391	0.0358
Socio-professional status ^1^	−0.2508	0.1124	−2.2314	0.0288

IPAQ–International Physical Activity Questionnaire; SE—standard error. ^1^ Employed vs. unemployed.

**Table 10:** Univariate linear regression analysis assessing the relationship between total physical activity [counts/minute] measured by the ActiGraph and selected variables in the total study population (*n* = 75).

	β	SE	t	*p*
Sex ^1^	−0.3499	0.1096	−3.1918	0.0021
Age [years]	−0.2024	0.1146	−1.7658	0.0816
Weight [kg]	−0.5019	0.1012	−4.9575	<0.0001
Place of living ^2^	−0.0068	0.1170	−0.0579	0.9539
Family situation ^3^	−0.1508	0.1157	−1.3035	0.1965
Education ^4^	−0.0121	0.1170	−0.1032	0.9181
Socio-professional status ^5^	−0.1306	0.1160	−1.1254	0.2641
Alcoholic drinks [units/week]	−0.0459	0.1169	−0.3928	0.6956
HAM–D [points]	0.1135	0.1163	0.9758	0.3324
MoCA [points]	0.2834	0.1122	2.5249	0.0137
RMR [kcal/d]	−0.4672	0.1035	−4.5151	<0.0001

HAM–D—Hamilton Depression Rating Scale; MoCA—Montreal Cognitive Assessment; RMR—resting metabolic rate; SE—standard error. ^1^ Men vs. women, ^2^ city vs. village, ^3^ in a relationship vs. single, ^4^ higher education vs. secondary + primary education, ^5^ employed vs. unemployed.

**Table 11:** Multivariate linear regression analysis assessing the relationship between total physical activity [counts/minute] measured by the ActiGraph and selected variables in the total study population (*n* = 75).

	β	SE	t	*p*
Sex ^1^	−0.1271	0.1264	−1.0055	0.3182
Age [years]	−0.0528	0.1111	−0.4750	0.6363
Weight [kg]	−0.3812	0.1704	−2.2377	0.0285
MoCA [points]	0.2423	0.1099	2.2040	0.0309
RMR [kcal/d]	−0.0703	0.1941	−0.3623	0.7183

EE—energy expenditure; MoCA—Montreal Cognitive Assessment; RMR—resting metabolic rate; SE—standard error. ^1^ Men vs. women.

**Table 12:** Univariate linear regression analysis assessing the relationship between MoCA points and selected variables in the total study population (*n* = 75).

	β	SE	t	*p*
Sex ^1^	−0.0778	0.1167	−0.6666	0.5071
Age [years]	−0.4480	0.1046	−4.2809	0.0001
Weight [kg]	−0.0469	0.1169	−0.4008	0.6897
Place of living ^2^	−0.0343	0.1170	−0.2929	0.7705
Family situation ^3^	0.0888	0.1166	0.7617	0.4487
Education ^4^	0.4279	0.1058	4.0447	0.0001
Socio-professional status ^5^	0.2158	0.1143	1.8881	0.0630
Alcoholic drinks [units/week]	0.0928	0.1165	0.7961	0.4286
HAM–D [points]	−0.1002	0.1165	−0.8608	0.3922
Total physical activity [MET—min/day]	−0.0781	0.1167	−0.6695	0.5053
RMR [kcal/d]	−0.0319	0.1170	−0.2727	0.7859
Total physical activity [counts/min]	0.2834	0.1122	2.5249	0.0137

HAM–D—Hamilton Depression Rating Scale; MoCA—Montreal Cognitive Assessment; RMR—resting metabolic rate; SE—standard error.^1^ Men vs. women, ^2^ city vs. village, ^3^ in a relationship vs. single, ^4^ higher education vs. secondary + primary education, ^5^ employed vs. unemployed.

**Table 13:** Multivariate linear regression analysis assessing the relationship between MoCA points and selected variables in the total study population (*n* = 75).

	β	SE	t	*p*
Age [years]	−0.3336	0.1115	−2.9909	0.0038
Education ^1^	0.3644	0.1022	3.5656	0.0007
Socio-professional status ^2^	−0.0275	0.1141	−0.2411	0.8102
Total physical activity [counts/min]	0.2167	0.1008	2.1505	0.0350 0.1400

HAM–D—Hamilton Depression Rating Scale; MoCA—Montreal Cognitive Assessment; RMR—resting metabolic rate; SE—standard error, ^1^ higher education vs. secondary + primary education, ^2^ employed vs. unemployed.

## Data Availability

The data presented in this study are available on request from the corresponding author. The data are not publicly available due to the disagreement of the study participants.

## Supplementary Materials

The following are available online at https://www.mdpi.com/article/10.3390/ijerph18158042/s1, Figure S1: Bland–Altman plot for sedentary behaviours [min/day] measured by the ActiGraph and IPAQ in subjects with MCI (*n* = 27), Figure S2: Bland–Altman plot for moderate activity [min/day] measured by the ActiGraph and IPAQ in subjects with MCI (*n* = 27), Figure S3: Bland–Altman plot for activity kilocalories per day measured by the ActiGraph and IPAQ in subject with MCI (*n* = 27), Figure S4: Bland–Altman plot for sedentary behaviours [min/day] measured by the ActiGraph and IPAQ in subjects with NCF (*n* = 48), Figure S5: Bland–Altman plot for moderate activity [min/day] measured by the ActiGraph and IPAQ in subjects with NCF (*n* = 48), Figure S6. Bland–Altman plot for activity kilocalories per day measured by the ActiGraph and IPAQ in subjects with NCF (*n* = 48). Table S1: Kappa (κ) coefficients between the tertiles of the objective and subjective method for measure physical activity in subjects with MCI (*n* = 27). Table S2: Kappa (κ) coefficients between the tertiles of the objective and subjective method for measure physical activity in subjects with NCF (*n* = 48). Table S3: Kendall’s tau-b coefficients between the tertiles objective and subjective method for measure physical activity in subjects with MCI (*n* = 27). Table S4: Kendall’s tau-b coefficients between the tertiles objective and subjective method for measure physical activity subjects with NCF (*n* = 48). Table S5: Univariate linear regression analysis assessing the relationship between total physical activity [MET—min/day] measured by IPAQ and selected variables in subjects with MCI (*n* = 27), Table S6: Univariate linear regression analysis assessing the relationship between total physical activity [counts/minute] measured by the ActiGraph and selected variables in subjects with MCI (*n* = 27), Table S7: Multivariate linear regression analysis assessing the relationship between total physical activity [counts/min] measured by the ActiGraph and selected variables in subjects with MCI (*n* = 27). Table S8: Univariate linear regression analysis assessing the relationship between total physical activity [MET—min/day] measured by IPAQ and selected variables in subjects with NCF (*n* = 48), Table S9: Univariate linear regression analysis assessing the relationship between total physical activity [counts/minute] measured by the ActiGraph and selected variables in subjects with NCF (*n* = 48), Table S10: Multivariate linear regression analysis assessing the relationship between total physical activity [counts/minute] measured by the ActiGraph and selected variables in subjects with NCF (*n* = 48). Table S11. Univariate linear regression analysis assessing the relationship between MoCA points and selected variables in the MCI group (*n* = 27). Table S12. Multivariate linear regression analysis assessing the relationship between MoCA points and selected variables in the MCI group (*n* = 27). Table S13. Univariate linear regression analysis assessing the relationship between MoCA points and selected variables in the NCF group (*n* = 48). Table S14. Multivariate linear regression analysis assessing the relationship between MoCA points and selected variables in the NCF group (*n* = 48).

## References

[1] Caspersen C.J., Powell K.E., Christenson G.M. (1985). Physical activity, exercise, and physical fitness: Definitions and distinctions for health-related research. Public Health Rep..

[2] Herzig K.H., Ahola R., Leppäluoto J., Jokelainen J., Jämsä T., Keinänen-Kiukaanniemi S. (2014). Light physical activity determined by a motion sensor decreases insulin resistance, improves lipid homeostasis and reduces visceral fat in high-risk subjects: PreDiabEx study RCT. Int. J. Obes..

[3] World Health Organization, Public Health Agency of Canada (2005). Preventing Chronic Diseases: A Vital Investment.

[4] World Health Organization (2007). A Guide for Population–Based Approaches to Increasing Levels of Physical Activity: Implementation of the WHO Global Strategy on Diet, Physical Activity and Health.

[5] World Health Organization (2008). The Global Burden of Disease: 2004 Update.

[6] World Health Organization (2009). Global Health Risks: Mortality and Burden of Disease Attributable to Selected Major Risks.

[7] Yaffe K., Barnes D., Nevitt M., Lui L.Y., Covinsky K. (2001). A prospective study of physical activity and cognitive decline in elderly women: Women who walk. Arch. Intern. Med..

[8] Laurin D., Verreault R., Lindsay J., MacPherson K., Rockwood K. (2001). Physical activity and risk of cognitive impairment and dementia in elderly persons. Arch. Neurol..

[9] Petersen R.C., Smith G.E., Waring S.C., Ivnik R.J., Kokmen E., Tagelos E.G. (1997). Aging, memory, and mild cognitive impairment. Int. Psychogeriatr..

[10] Kivipelto M., Ngandu T., Laatikainen T., Winblad B., Soininen H., Tuomilehto J. (2006). Risk score for the prediction of dementia risk in 20 years among middle aged people: A longitudinal, population-based study. Lancet Neurol..

[11] Manly J.J., Tang M.X., Schupf N., Stern Y., Vonsattel J.P., Mayeux R. (2008). Frequency and course of mild cognitive impairment in a multiethnic community. Ann. Neurol..

[12] Ismail Z., Elbayoumi H., Fischer C.E., Hogan D.B., Millikin C.P., Schweizer T., Mortby M.E., Smith E.E., Patten S.B., Fiest K.M. (2017). Prevalence of Depression in Patients with Mild Cognitive Impairment: A Systematic Review and Meta-analysis. JAMA Psychiatry.

[13] Langa K.M., Levine D.A. (2014). The diagnosis and management of mild cognitive impairment: A clinical review. JAMA.

[14] Bull F.C., Al-Ansari S.S., Biddle S., Borodulin K., Buman M.P., Cardon G., Carty C., Chaput J.-P., Chastin S., Chou R. (2020). World Health Organization 2020 guidelines on physical activity and sedentary behaviour. Br. J. Sports Med..

[15] Ainsworth B.M. (2008). How do I measure physical activity in my patients? Questionnaires and objective methods. Br. J. Sports Med..

[16] Biernat E., Strupnicki R. (2005). An overview of internationally applicable questionnaires designed for assessing physical activity. Phys. Educ. Sport.

[17] Hagströmer M., Pekka O., Sjöström M. (2006). The International Physical Activity Questionnaire (IPAQ): A study of concurrent and construct validity. Public Health Nutr..

[18] International Physical Activity Questionnaire. https://sites.google.com/site/theipaq/.

[19] Craig C.L., Marshall A.L., Sjöström M., Bauman A.E., Booth M.L., Ainsworth B.E., Pratt M., Ekelund U., Yngve A., Sallis J.F. (2003). International physical activity questionnaire: 12-country reliability and validity. Med. Sci. Sports Exerc..

[20] Sjostrom M., Ekelund U., Poortvliet E. (2000). Assessment of physical activity using IPAQ (version 4) and activity monitors (CSA). Meas. Phys. Educ. Exerc. Sci..

[21] Prince S.A., Adamo K.B., Hamel M.E., Hardt J., Connor Gorber S., Tremblay M. (2008). A comparison of direct versus self–report measures for assessing physical activity in adults: A systematic review. Int. J. Behav. Nutr. Phys. Act..

[22] Shiroma E.J., Schepps M.A., Harezlak J., Chen K.Y., Matthews C.E., Koster A., Caserotti P., Glynn N.W., Harris T.B. (2016). Daily physical activity patterns from hip- and wrist-worn accelerometers. Physiol. Meas..

[23] Hildebrand M., Van Hees V.T., Hansen B.H., Ekelund U. (2014). Age group comparability of raw accelerometer output from wrist- and hip-worn monitors. Med. Sci. Sports Exerc..

[24] Curry W.B., Thompson J.L. (2015). Comparability of accelerometer- and IPAQ-derived physical activity and sedentary time in South Asian women: A cross-sectional study. Eur. J. Sport Sci..

[25] Lipert A., Jegier A. (2017). Comparison of different physical activity measurement methods in adults aged 45 to 64 years under free–living conditions. Clin. J. Sport Med..

[26] Innerd P., Catt M., Collerton J., Davies K., Trenell M., Kirkwood T.B., Jagger C. (2015). A comparison of subjective and objective measures of physical activity from the Newcastle 85+ study. Age Ageing.

[27] Colley R.C., Butler G., Garriguet D., Prince S.A., Roberts K.C. (2019). Comparison of self-reported and accelerometer-measured physical activity among Canadian youth. Health Rep..

[28] Ahmad M.H., Salleh R., Nor N.S.M., Baharuddin A., Hasani W.S.R., Omar A., Jamil A.T., Appukutty M., Wan Muda W.A.M., Aris T. (2018). Comparison between self-reported physical activity (IPAQ-SF) and pedometer among overweight and obese women in the MyBFF@home study. BMC Womens Health.

[29] Liu S.H., Eaton C.B., Driban J.B., McAlindon T.E., Lapane K.L. (2016). Comparison of self-report and objective measures of physical activity in US adults with osteoarthritis. Rheumatol. Int..

[30] O’Neill B., McDonough S.M., Wilson J.J., Bradbury I., Hayes K., Kirk A., Kent L., Cosgrove D., Bradley J.M., Tully M.A. (2017). Comparing accelerometer, pedometer and a questionnaire for measuring physical activity in bronchiectasis: A validity and feasibility study?. Respir. Res..

[31] Rosenbaum S., Tiedemann A., Sherrington C., van der Ploeg H.P. (2014). Assessing physical activity in people with posttraumatic stress disorder: Feasibility and concurrent validity of the International Physical Activity Questionnaire—Short form and actigraph accelerometers. BMC Res. Notes.

[32] Magierska J., Magierski R., Fendler W., Kłoszewska I., Sobów T.M. (2012). Clinical application of the Polish adaptation of the Montreal Cognitive Assessment (MoCA) test in sreening for cognitive impairment. Neurol. Neurochir. Pol..

[33] Von Elm E., Altman D.G., Egger M., Pocock S.J., Gøtzsche P.C., Vandenbroucke J.P. (2008). The strengthening the reporting of observational studies in epidemiology (STROBE) statement: Guidelines for reporting observational studies. J. Clin. Epidemiol..

[34] Biernat E. (2013). International Physical Activity Questionnaire—Polish long version. Pol. J. Sport Med..

[35] Leavitt M.O. (2008). 2008 Physical Activity Guidelines for Americans.

[36] Hills A.P., Mokhtar N., Byrne N.M. (2014). Assessment of physical activity and energy expenditure: An overview of objective measures. Front. Nutr..

[37] Freedson P.S., Melanson E., Sirard J. (1998). Calibration of the computer science and applications, Inc. accelerometer. Med. Sci. Sports Exerc..

[38] Ciesielska N., Sokołowski R., Mazur E., Podhorecka M., Polak-Szabela A., Kędziora-Kornatowska K. (2016). Is the Montreal Cognitive Assessment (MoCA) test better suited than the Mini-Mental State Examination (MMSE) in mild cognitive impairment (MCI) dtection among people aged over 60? Meta-Analysis. Psychiatr. Pol..

[39] Hagstromer M., Ainsworth B.E., Oja P., Sjostrom M. (2010). Comparison of a subjective and an objective measure of physical activity in a population sample. J. Phys. Act. Health.

[40] Bland J.M., Altman D.G. (1999). Measuring agreement in method comparison studies. Stat. Methods Med. Res..

[41] Oyeyemi A.L., Umar M., Oguche F., Aliyu S.U., Oyeyemi A.Y. (2014). Accelerometer-determined physical activity and its comparison with the International Physical Activity Questionnaire in a sample of Nigerian adults. PLoS ONE.

[42] Northey J.M., Cherbuin N., Pumpa K.L., Smee D.J., Rattray B. (2018). Exercise interventions for cognitive function in adults older than 50: A systematic review with meta-analysis. Br. J. Sports Med..

[43] Sofi F., Valecchi D., Bacci D., Abbate R., Gensini G.F., Casini A., Macchi C. (2011). Physical activity and risk of cognitive decline: A meta–analysis of prospective studies. J. Intern. Med..

[44] Van der Wardt V., Hancox J.E., Burgon C., Bajwa R., Goldberg S., Harwood R.H. (2020). Measuring physical activity levels in people with mild cognitive impairment or mild dementia. J. Aging Phys. Act..

[45] Ekelund U., Sepp H., Brage S., Becker W., Jakes R., Hennings M., Wareham N.J. (2006). Criterion-related validity of the last 7-day, short form of the International Physical Activity Questionnaire in Swedish adults. Public Health Nutr..

[46] Matthews C.E., Ainsworth B.E., Hanby C., Pate R.R., Addy C., Freedson P.S., Jones D.A., Macera C.A. (2005). Development and testing of a short physical activity recall questionnaire. Med. Sci. Sports Exerc..

[47] Skender S., Ose J., Chang-Claude J., Paskow M., Brühmann B., Siegel E.M., Steindorf K., Ulrich C.M. (2016). Accelerometry and physical activity questionnaires—A systematic review. BMC Public Health.

[48] Sabia S., van Hees V.T., Shipley M.J., Trenell M.I., Hagger-Johnson G., Elbaz A., Kivimaki M., Singh-Manoux A. (2014). Association between questionnaire- and accelerometer-assessed physical activity: The role of sociodemographic factors. Am. J. Epidemiol..

[49] Sirard J.R., Pate R.R. (2001). Physical activity assessment in children and adolescents. Sports Med..

[50] Ham S.A., Reis J.P., Strath S.J., Dubose K.D., Ainsworth B.E. (2007). Discrepancies between methods of identifying objectively determined physical activity. Med. Sci. Sports Exerc..

[51] Leinonen A.M., Ahola R., Kulmala J., Hakonen H., Vähä-Ypyä H., Herzig K.H., Auvinen J., Keinänen-Kiukaanniemi S., Sievänen H., Tammelin T.H. (2017). Measuring physical activity in free-living conditions-comparison of three accelerometry-based methods. Front. Physiol..

[52] Tudor-Locke C., Barreira T.V., Schuna J.M. (2015). Comparison of step outputs for waist and wrist accelerometer attachment sites. Med. Sci. Sports Exerc..

[53] Brown W., Bauman A., Chey T., Trost S., Mummery K. (2004). Comparison of surveys used to measure physical activity. Aust. N. Z. J. Public Health.

[54] Chapie A., Arena S. (2020). Benefits and barriers of activity trackers among older adults. Home Healthc. Now.

[55] Kononova A., Li L., Kamp K., Bowen M., Rikard R.V., Cotton S., Peng W. (2019). The Use of Wearable Activity Trackers Among Older Adults: Focus Group Study of Tracker Perceptions, Motivators, and Barriers in the Maintenance Stage of Behavior Change. JMIR mHealth uHealth.

[56] Kawagoshi A., Kiyokawa N., Sugawara K., Takahashi H., Sakata S., Satake M., Shioya T. (2015). Effects of low-intensity exercise and home-based pulmonary rehabilitation with pedometer feedback on physical activity in elderly patients with chronic obstructive pulmonary disease. Respir. Med..

[57] Cooper C., Gross A., Brinkman C., Pope R., Allen K., Hastings S., Bogen B.E., Goode A.P. (2018). The impact of wearable motion sensing technology on physical activity in older adults. Exp. Gerontol..

[58] Link-Age Connect 2019 Technology Survey Older Adults Age 55–100. https://linkageconnect.com/wp-content/uploads/2019/05/2019-Link-age-Connect-Technology-Study-Report.pdf.

[59] Petersen R.C., Lopez O., Armstrong M.J., Getchius T.S.D., Ganguli M., Gloss D., Gronseth G.S., Marson D., Pringsheim T., Day G.S. (2018). Practice guideline update summary: Mild cognitive impairment: Report of the Guideline Development, Dissemination, and Implementation Subcommittee of the American Academy of Neurology. Neurology.

[60] Hussin N.M., Shahar S., Yahya H.M., Din N.C., Singh D.K.A., Omar M.A. (2019). Incidence and predictors of mild cognitive impairment (MCI) within a multi-ethnic Asian populace: A community-based longitudinal study. BMC Public Health.

[61] Niu H., Álvarez-Álvarez I., Guillén-Grima F., Aguinaga-Ontoso I. (2017). Prevalence and incidence of Alzheimer’s disease in Europe: A meta-analysis. Neurologia.

[62] Filley C.M. (1997). Alzheimer’s disease in women. Am. J. Obstet. Gynecol..

[63] Sallis J.F., Prochaska J.J., Taylor W.C. (2000). A review of correlates of physical activity of children and adolescents. Med. Sci. Sports Exerc..

[64] Mitáš J., Cerin E., Reis R.S., Conway T.L., Cain K.L., Adams M.A., Schofield G., Sarmiento O.L., Christiansen L.B., Davey R. (2019). Do associations of sex, age and education with transport and leisure-time physical activity differ across 17 cities in 12 countries?. Int. J. Behav. Nutr. Phys. Act..

[65] Valkenet K., Veenhof C. (2019). Validity of three accelerometers to investigate lying, sitting, standing and walking. PLoS ONE.

[66] John D., Freedson P. (2012). ActiGraph and Actical physical activity monitors: A peek under the hood. Med. Sci. Sports Exerc..

[67] Panza F., Frisardi V., Capurso C., D’Introno A., Colacicco A.M., Imbimbo B.P., Santamato A., Vendemiale G., Seripa D., Pilotto A. (2010). Late-life depression, mild cognitive impairment, and dementia: Possible continuum?. Am. J. Geriatr. Psychiatry.

[68] Steffens D.C. (2012). Depressive symptoms and mild cognitive impairment in the elderly: An ominous combination. Biol. Psychiatry.

[69] Luck T., Luppa M., Wiese B., Maier W., van den Bussche H., Eisele M., Jessen F., Weeg D., Weyerer S., Pentzek M. (2012). Prediction of incident dementia: Impact of impairment in instrumental activities of daily living and mild cognitive impairment-results from the German study on ageing, cognition, and dementia in primary care patients. Am. J. Geriatr. Psychiatry.

[70] Heerman W.J., Bennett W.L., Kraschnewski J.L., Nauman E., Staiano A.E., Wallston K.A. (2018). Willingness to participate in weight-related research as reported by patients in PCORnet clinical data research networks. BMC Obes..

[71] Arguello D., Andersen K., Morton A., Freedson P.S., Intille S.S., John D. (2018). Validity of proximity sensor-based wear-time detection using the ActiGraph GT9X. J. Sports Sci..

[72] Montoye A.H.K., Pivarnik J.M., Mudd L.M., Biswas S., Pfeiffer K.A. (2016). Validation and comparison of accelerometers worn on the hip, thigh, and wrists for measuring physical activity and sedentary behavior. AIMS Public Health.

