# Supplementary material for: Comparison of Subjective and Objective Methods to Measure the Physical Activity of Non-Depressed Middle-Aged Healthy Subjects with Normal Cognitive Function and Mild Cognitive Impairment—A Cross-Sectional Study
Source: Int J Environ Res Public Health. 2021 Jul 29;18(15):8042. doi: 10.3390/ijerph18158042 (PMC8345702; doi:10.3390/ijerph18158042)
Supplement: Supplementary file 1 [file ijerph-18-08042-s001.zip › Supplementary Figures 2021-07-28.pdf]

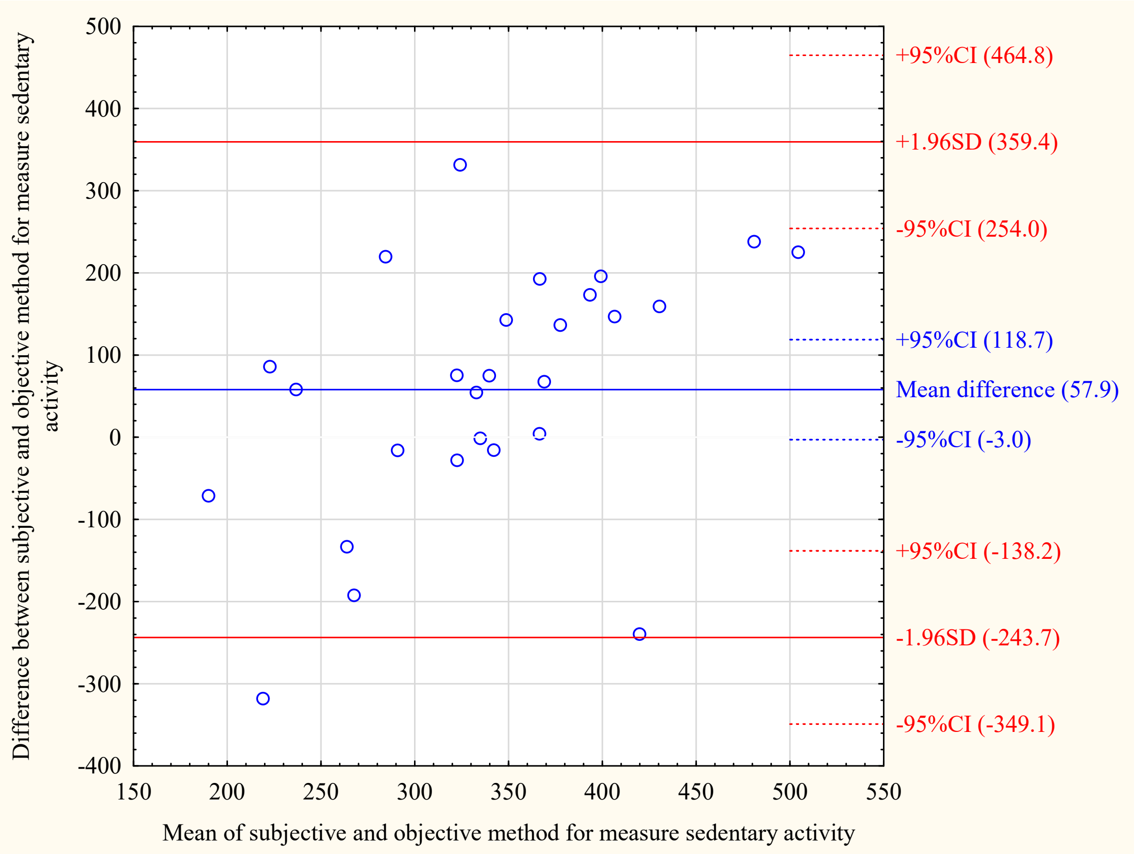

Figure S1. Bland-Altman plot for sedentary behaviour [min/day] measured by the IPAQ and ActiGraph in subjects with MCI (*n* = 27).

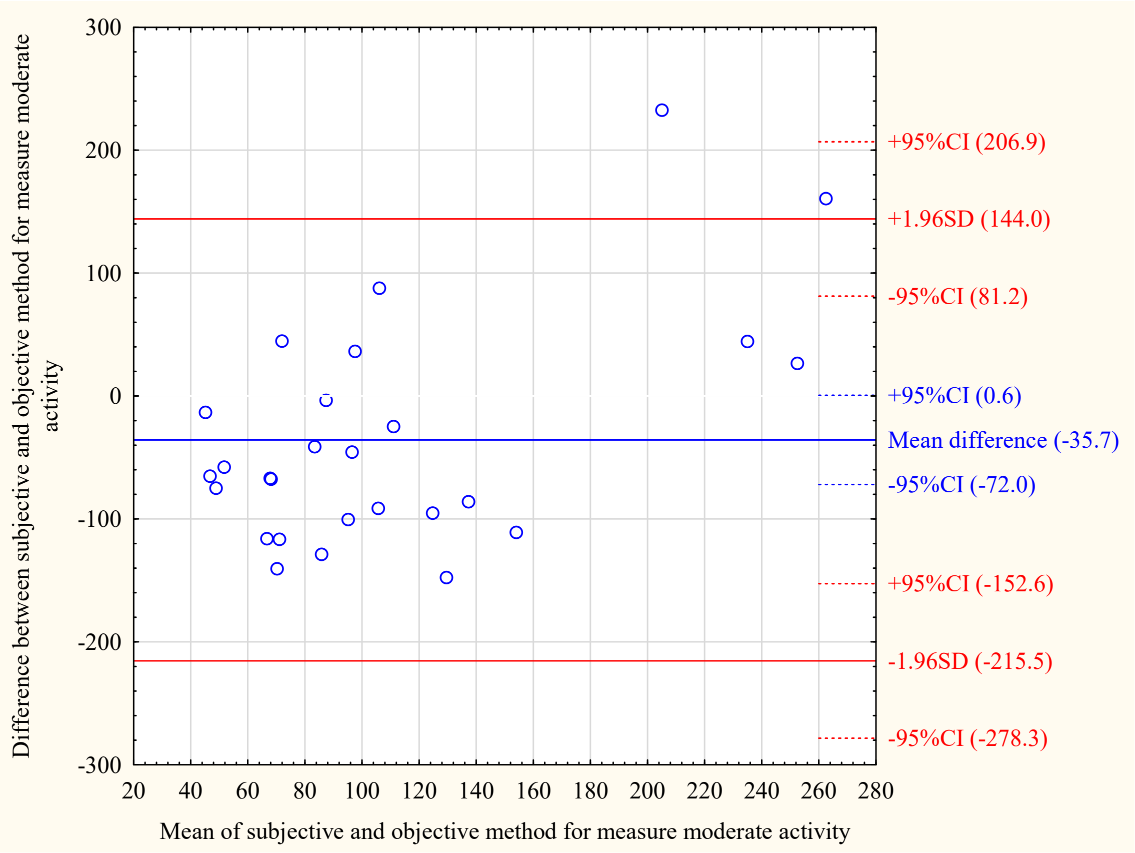

Figure S2. Bland-Altman plot for moderate activity [min/day] measured by the IPAQ and ActiGraph in subjects with MCI (*n* = 27).

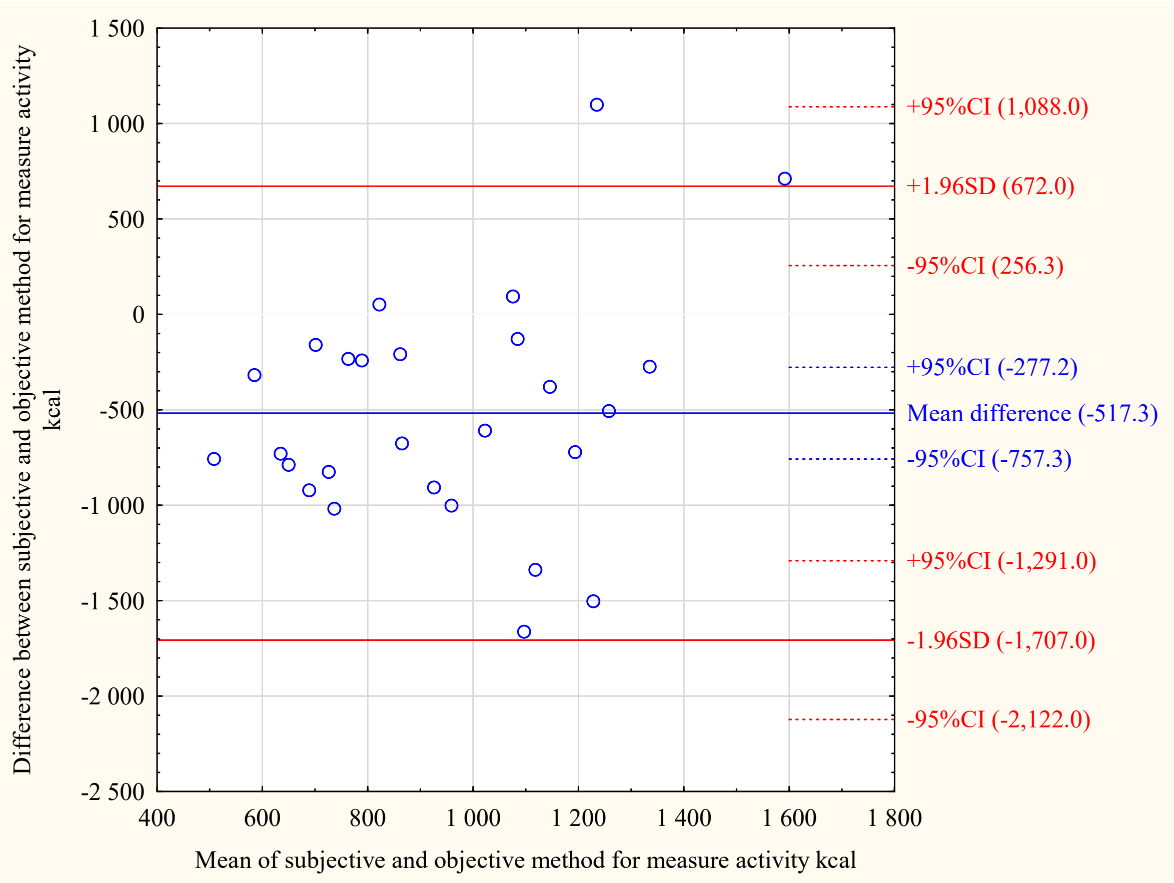

Figure S3. Bland-Altman plot for activity kilocalories per day measured by the IPAQ and ActiGraph in subject with MCI (*n* = 27).

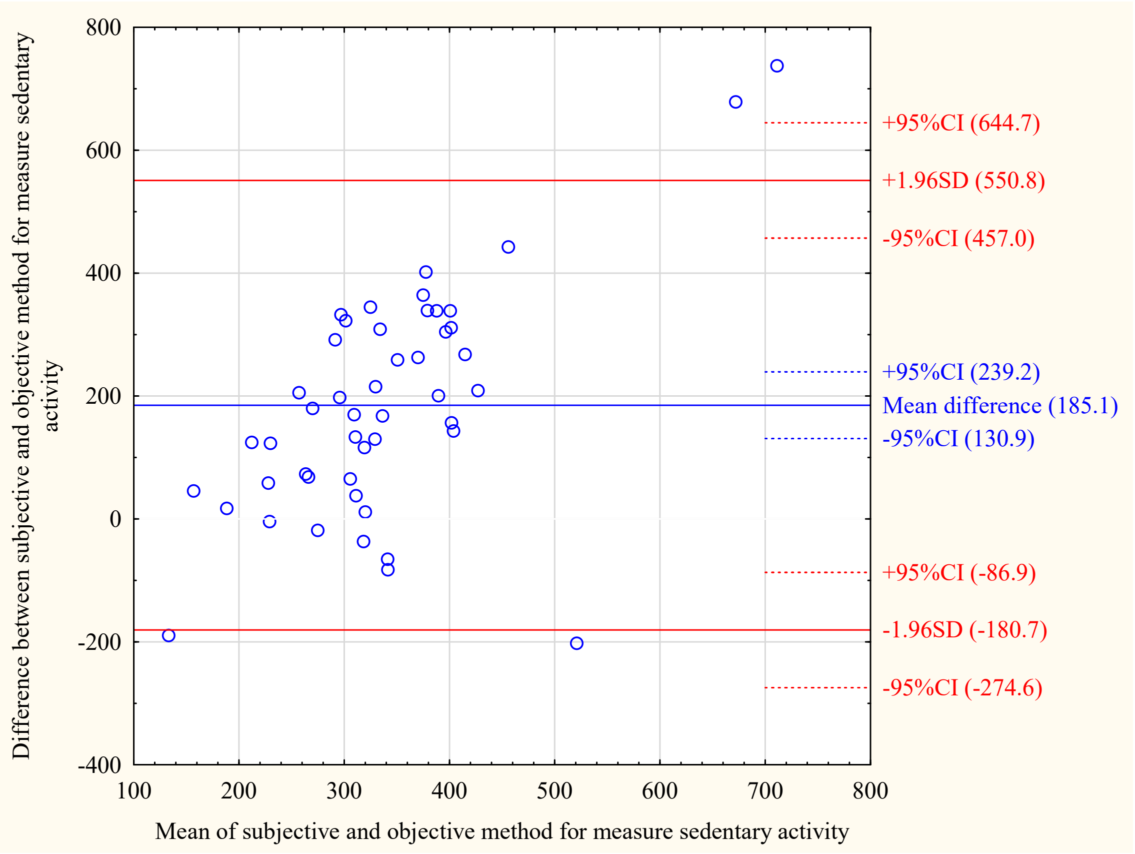

Figure S4. Bland-Altman plot for sedentary behaviour [min/day] measured by the IPAQ and ActiGraph in subjects with NCF ($n = 48$).

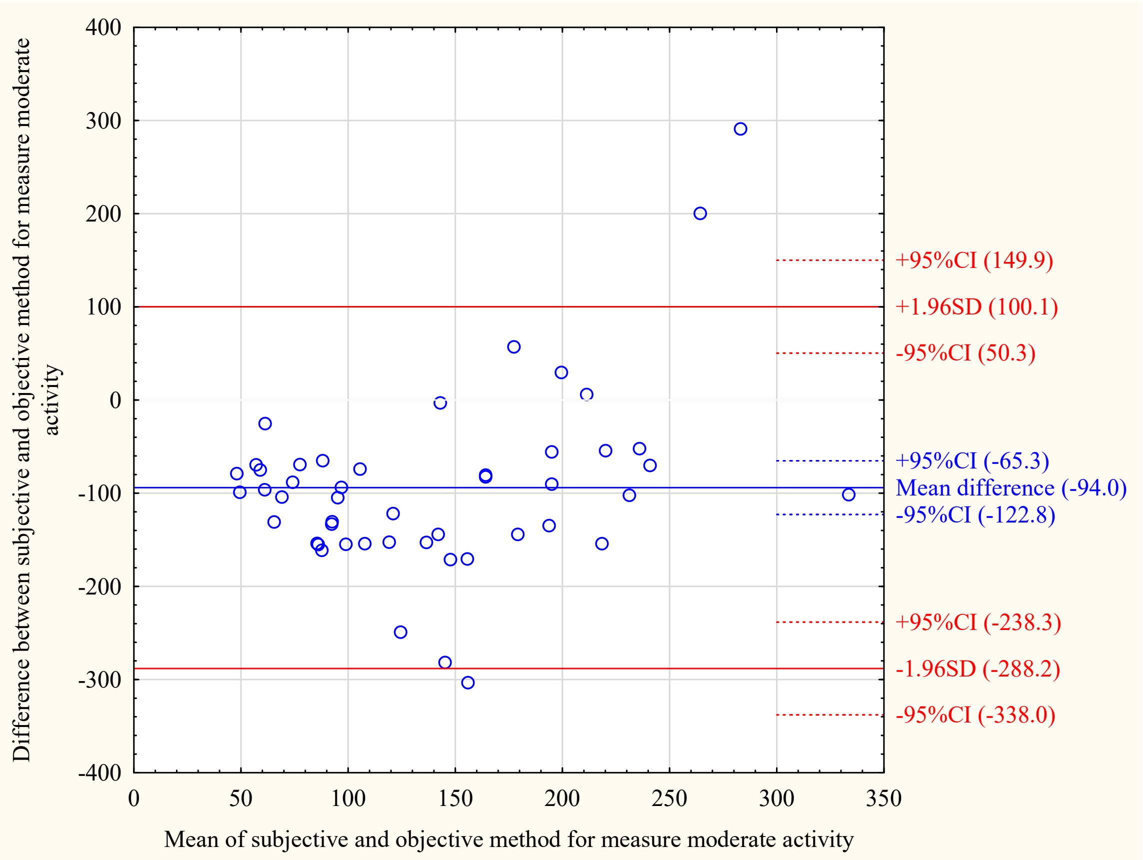

Figure S5. Bland-Altman plot for moderate activity [min/day] measured by the IPAQ and ActiGraph in subjects with NCF ($n = 48$).

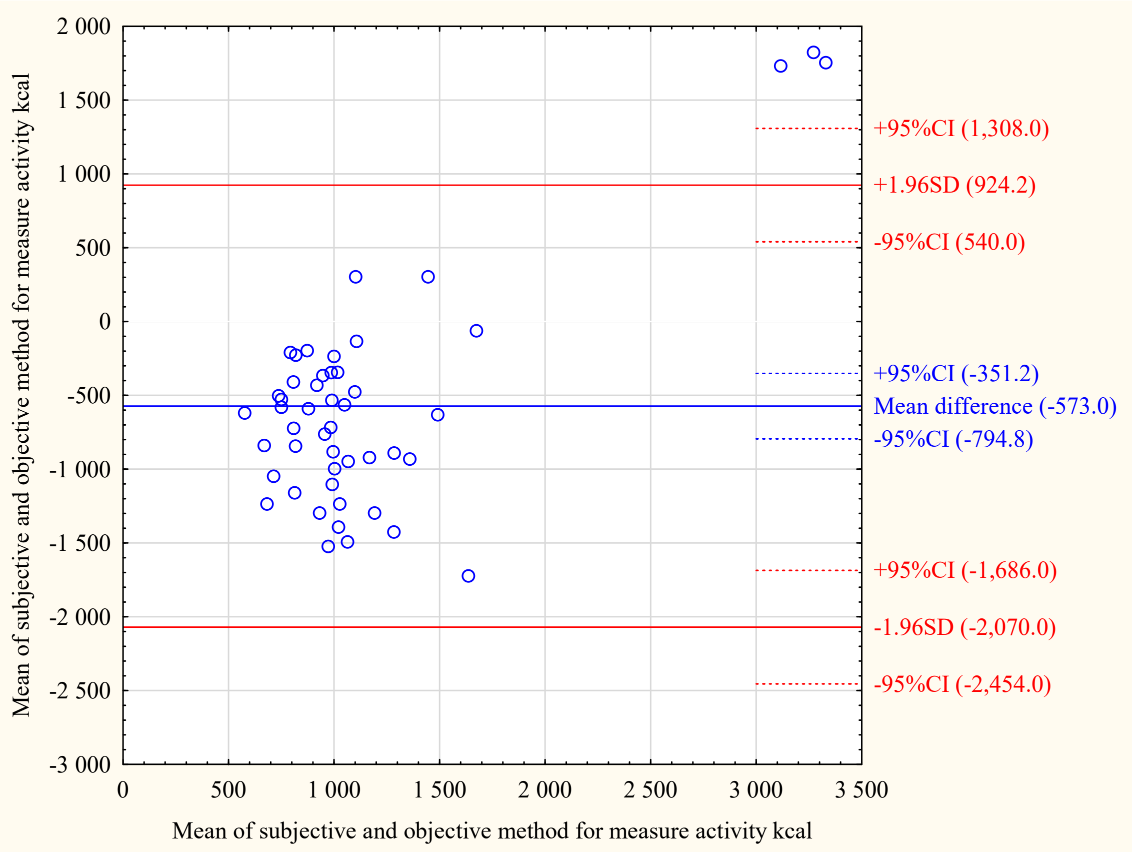

Figure S6. Bland-Altman plot for activity kilocalories per day measured by the IPAQ and ActiGraph in subjects with NCF ($n = 48$).
