# Supplementary material for: Comparison of Subjective and Objective Methods to Measure the Physical Activity of Non-Depressed Middle-Aged Healthy Subjects with Normal Cognitive Function and Mild Cognitive Impairment—A Cross-Sectional Study
Source: Int J Environ Res Public Health. 2021 Jul 29;18(15):8042. doi: 10.3390/ijerph18158042 (PMC8345702; doi:10.3390/ijerph18158042)
Supplement: Supplementary file 1 [file ijerph-18-08042-s001.zip › Supplementary Tables - revision 2021-07-28.pdf]

Table S1. Kappa (κ) coefficients between the tertiles of the objective and subjective method for measure physical activity in subjects with MCI (*n* = 27).

	**κ (95% CI)**	**SE**	**Z**	***p***
Total physical activity[1]	0.17 (-0.20 – 0.55)	0.19	0.91	0.3641
Sedentary behaviour[2]	-0.06 (-0.33 – 0.45)	0.19	0.32	0.7525
Moderate activity[3]	0.17 (-0.23 – 0.57)	0.19	0.89	0.3720
Kcal/day[4]	0.17 (-0.18 – 0.52)	0.19	0.87	0.3845

[1]Total physical activity [counts/minute] measured by the ActiGraph *vs*. total physical activity [MET min/day] measured by IPAQ

[2]Sedentary behaviour [min/day] measured by the ActiGraph *vs*. IPAQ

[3]Moderate activity [min/day] measured by the ActiGraph *vs*. IPAQ

[4]Activity kilocalories per day measured by the ActiGraph *vs*. IPAQ

Table S2. Kappa (κ) coefficients between the tertiles of the objective and subjective method for measure physical activity in subjects with NCF (*n* = 48).

	**κ (95% CI)**	**SE**	**Z**	***p***
Total physical activity[1]	0.26 (-0.02 – 0.54)	0.14	1.78	0.0743
Sedentary behaviour[2]	-0.03 (-0.32 – 0.25)	0.14	-0.22	0.8258
Moderate activity[3]	0.35 (0.10 – 0.61)	0.14	2.46	0.0139
Kcal/day[4]	0.10 (-0.19 – 0.39)	0.14	0.69	0.4888

[1]Total physical activity [counts/minute] measured by the ActiGraph *vs*. total physical activity [MET min/day] measured by IPAQ

[2]Sedentary behaviour [min/day] measured by the ActiGraph *vs*. IPAQ

[3]Moderate activity [min/day] measured by the ActiGraph *vs*. IPAQ

[4]Activity kilocalories per day measured by the ActiGraph *vs*. IPAQ

Table S3. Kendall's tau-b coefficients between the tertiles objective and subjective method for measure physical activity in subjects with MCI (n = 27).

	**Kendall's tau-b**	***p***
Total physical activity[1]	0.1322	0.3333
Sedentary behaviour[2]	0.0539	0.6931
Moderate activity[3]	0.1618	0.2364
Kcal/day[4]	0.1440	0.2919

[1]Total physical activity [counts/minute] measured by the ActiGraph *vs*. total physical activity [MET min/day] measured by IPAQ

[2]Sedentary behaviour [min/day] measured by the ActiGraph *vs*. IPAQ

[3]Moderate activity [min/day] measured by the ActiGraph *vs*. IPAQ

[4]Activity kilocalories per day measured by the ActiGraph *vs*. IPAQ

Table S4. Kendall's tau-b coefficients between the tertiles objective and subjective method for measure physical activity subjects with NCF (n = 48).

	**Kendall's tau-b**	***p***
Total physical activity[1]	0.2269	0.0229
Sedentary behaviour[2]	-0.0274	0.7838
Moderate activity[3]	0.3183	0.0014
Kcal/day[4]	0.0875	0.3802

[1]Total physical activity [counts/minute] measured by the ActiGraph *vs*. total physical activity [MET min/day] measured by IPAQ

[2]Sedentary behaviour [min/day] measured by the ActiGraph *vs*. IPAQ

[3]Moderate activity [min/day] measured by the ActiGraph *vs*. IPAQ

[4]Activity kilocalories per day measured by the ActiGraph *vs*. IPAQ

Table S5. Univariate linear regression analysis assessing the relationship between total physical activity [MET-min/day] measured by IPAQ and selected variables in subjects with MCI (n = 27).

	**β**	**SE**	**t**	***p***
Sex[1]	-0.0978	0.1990	-0.4915	0.6273
Age [years]	-0.0209	0.2000	-0.1048	0.9174
Weight [kg]	-0.4071	0.1827	-2.2287	0.0351
Place of living[2]	0.1610	0.1974	0.8159	0.4223
Family situation[3]	0.2453	0.1939	1.2653	0.2174
Education[4]	-0.1613	0.1974	-0.8170	0.4217
Socio-professional status[5]	-0.3030	0.1906	-1.5896	0.1245
Alcoholic drinks [units/week]	0.1893	0.1964	0.9641	0.3442
HAM-D [points]	-0.1214	0.1985	-0.6115	0.5464
MoCA [points]	-0.2452	0.1939	-1.2645	0.2177
RMR [kcal/d]	-0.2352	0.1944	-1.2097	0.2377

HAM-D – Hamilton Depression Rating Scale; IPAQ – International Physical Activity Questionnaire; MCI – Mild Cognitive Impairment; MoCA – Montreal Cognitive; RMR – Resting Metabolic Rate; SE – Standard Error

[1]Men *vs*. women

[2]City *vs*. village

[3]In a relationship *vs*. single

[4]Higher education *vs*. secondary + primary education

[5]Employed *vs*. unemployed

Table S6. Univariate linear regression analysis assessing the relationship between total physical activity [counts/min] measured by the ActiGraph and selected variables in subjects with MCI ($n = 27$).

	**β**	**SE**	**t**	***p***
Sex[1]	-0.1130	0.1987	-0.5684	0.5748
Age [years]	-0.3730	0.1856	-2.0100	0.0553
Weight [kg]	-0.4835	0.1751	-2.7621	0.0106
Place of living[2]	0.3529	0.1871	1.8861	0.0709
Family situation[3]	0.1241	0.1985	0.6254	0.5374
Education[4]	-0.2696	0.1926	-1.3998	0.1739
Socio-professional status[5]	0.1397	0.1980	0.7054	0.4871
Alcoholic drinks [units/week]	0.2240	0.1949	1.1494	0.2613
HAM-D [points]	-0.3977	0.1835	-2.1672	0.0399
MoCA [points]	-0.1656	0.1972	-0.8394	0.4092
RMR [kcal/d]	-0.5573	0.1661	-3.3564	0.0025

HAM-D – Hamilton Depression Rating Scale; MCI – Mild Cognitive Impairment; MoCA – Montreal Cognitive Assessment; RMR – Resting Metabolic Rate; SE – Standard Error

[1]Men *vs*. women

[2]City *vs*. village

[3]In a relationship *vs*. single

[4]Higher education *vs*. secondary + primary education

[5]Employed *vs*. unemployed

Table S7. Multivariate linear regression analysis assessing the relationship between total physical activity [counts/min] measured by the ActiGraph and selected variables in subjects with MCI (*n* = 27).

	**β**	**SE**	**t**	***p***
Age [years]	0.0012	0.2599	0.0044	0.9965
Weight [kg]	0.0316	0.2501	0.1265	0.9006
Place of living[1]	-0.4002	0.2838	-1.4104	0.1731
HAM-D [points]	-0.0670	0.2064	-0.3246	0.7487
RMR [kcal/d]	-0.6050	0.2505	-2.4149	0.0249

HAM-D – Hamilton Depression Rating Scale; MCI – Mild Cognitive Impairment;RMR – Resting Metabolic Rate; SE – Standard Error

[1]City *vs*. village

Table S8. Univariate linear regression analysis assessing the relationship between total physical activity [MET-min/day] measured by IPAQ and selected variables in subjects with NCF ($n = 48$).

	**β**	**SE**	**t**	***p***
Sex[1]	-0.1022	0.1467	-0.6965	0.4896
Age [years]	0.2254	0.1436	1.5691	0.1235
Weight [kg]	0.2002	0.1445	1.3861	0.1724
Place of living[2]	0.0571	0.1472	0.3876	0.7001
Family situation[3]	0.0274	0.1474	0.1856	0.8536
Education[4]	-0.1523	0.1457	-1.0448	0.3016
Socio-professional status[5]	-0.1872	0.1448	-1.2928	0.2025
Alcoholic drinks [units/week]	0.2112	0.1441	1.4651	0.1497
HAM-D [points]	0.1875	0.1448	1.2947	0.2019
MoCA [points]	-0.3482	0.1382	-2.5189	0.0153
RMR [kcal/d]	0.2126	0.1441	1.4757	0.1468

HAM-D – Hamilton Depression Rating Scale; IPAQ – International Physical Activity Questionnaire; MoCA – Montreal Cognitive; NCF – Normal Cognitive Functions; RMR – Resting Metabolic Rate; SE – Standard Error

[1]Men *vs*. women

[2]City *vs*. village

[3]In a relationship *vs*. single

[4]Higher education *vs*. secondary + primary education

[5]Employed *vs*. unemployed

Table S9. Univariate linear regression analysis assessing the relationship between total physical activity [counts/min] measured by the ActiGraph and selected variables in subjects with NCF ($n = 48$).

	**β**	**SE**	**t**	***p***
Sex[1]	-0.3764	0.1366	-2.7556	0.0084
Age [years]	0.0416	0.1473	0.2822	0.7790
Weight [kg]	-0.5487	0.1233	-4.4510	0.0001
Place of living[2]	0.0891	0.1469	0.6070	0.5469
Family situation[3]	-0.2208	0.1438	-1.5351	0.1316
Education[4]	-0.1587	0.1456	-1.0904	0.2812
Socio-professional status[5]	-0.3007	0.1406	-2.1383	0.0378
Alcoholic drinks [units/week]	-0.1869	0.1448	-1.2907	0.2033
HAM-D [points]	0.4001	0.1351	2.9612	0.0048
MoCA [points]	-0.0771	0.1470	-0.5244	0.6025
RMR [kcal/d]	-0.4593	0.1310	-3.5065	0.0010

EE – Energy Expenditure; HAM-D – Hamilton Depression Rating Scale; MoCA – Montreal Cognitive Assessment; NCF – Normal Cognitive Functions; RMR – Resting Metabolic Rate; SE – Standard Error

[1]Men *vs*. women

[2]City *vs*. village

[3]In a relationship *vs*. single

[4]Higher education *vs*. secondary + primary education

[5]Employed *vs*. unemployed

Table S10. Multivariate linear regression analysis assessing the relationship between total physical activity [counts/min] measured by the ActiGraph and selected variables in subjects with NCF ($n = 48$).

	**β**	**SE**	**t**	***p***
Sex[1]	-0.0889	0.1587	-0.5600	0.5785
Weight [kg]	-0.6356	0.2062	-3.0817	0.0036
Socio-professional status[2]	-0.2931	0.1093	-2.6809	0.0104
HAM-D [points]	0.3204	0.1145	2.7980	0.0077
RMR [kcal/d]	0.2046	0.2475	0.8265	0.4132

HAM-D – Hamilton Depression Rating Scale; NCF – Normal Cognitive Functions; RMR – Resting Metabolic Rate; SE – Standard Error

[1]Men *vs*. women

[2]Employed *vs*. unemployed

Table S11. Univariate linear regression analysis assessing the relationship between MoCA points and selected variables in the MCI group ($n = 27$).

	**β**	**SE**	**t**	***p***
Sex[1]	0.7089	0.1411	5.0257	< 0.0001
Age [years]	-0.3903	0.1841	-2.1194	0.0442
Weight [kg]	0.1608	0.1974	0.8145	0.4230
Place of living[2]	-0.3809	0.1849	-2.0601	0.0499
Family situation[3]	0.4691	0.1766	2.6558	0.0136
Education[4]	0.5249	0.1702	3.0832	0.0049
Socio–professional status[5]	0.5202	0.1708	3.0455	0.0054
Alcoholic drinks [units/week]	0.3035	0.1906	1.5926	0.1238
HAM–D [points]	0.0077	0.2000	0.0383	0.9698
RMR [kcal/d]	0.3684	0.1859	1.9813	0.0587
Total physical activity [MET-min/day]	-0.2452	0.1939	-1.2645	0.2177
Total physical activity [counts/min]	-0.1656	0.1972	-0.8394	0.4092

HAM–D – Hamilton Depression Rating Scale; MoCA – Montreal Cognitive Assessment; RMR – Resting Metabolic Rate; SE – Standard Error

[1]Men *vs*. women

[2]City *vs*. village

[3]In a relationship *vs*. single

[4]Higher education *vs*. secondary + primary education

[5]Employed *vs*. unemployed

[6]At least good *vs*. lower than good

Table S12. Multivariate linear regression analysis assessing the relationship between MoCA points and selected variables in the MCI group ($n = 27$).

	**β**	**SE**	**t**	***p***
Sex[1]	0.9074	0.4637	1.9566	0.0653
Age [years]	-0.5973	0.4492	-1.3297	0.1994
Place of living[2]	0.4708	0.3960	1.1889	0.2491
Family situation[3]	-0.0369	0.2216	-0.1664	0.8696
Education[4]	-0.1146	0.2544	-0.4503	0.6576
Socio–professional status[5]	-0.0579	0.3018	-0.1918	0.8500
RMR [kcal/d]	0.0488	0.2039	0.2395	0.8133

HAM–D – Hamilton Depression Rating Scale; MoCA – Montreal Cognitive Assessment; RMR – Resting Metabolic Rate; SE – Standard Error[1]Men *vs*. women

[2]City *vs*. village

[3]In a relationship *vs*. single

[4]Higher education *vs*. secondary + primary education

[5]Employed *vs*. unemployed

Table S13. Univariate linear regression analysis assessing the relationship between MoCA points and selected variables in the NCF group (n = 48).

	**β**	**SE**	**t**	***p***
Sex[1]	-0.0788	0.1470	-0.5364	0.5943
Age [years]	-0.2465	0.1429	-1.7251	0.0912
Weight [kg]	-0.0486	0.1473	-0.3300	0.7429
Place of living[2]	-0.3074	0.1403	-2.1907	0.0336
Family situation[3]	0.2681	0.1420	1.8871	0.0655
Education[4]	-0.1780	0.1451	-1.2267	0.2262
Socio–professional status[5]	0.2282	0.1436	1.5893	0.1188
Alcoholic drinks [units/week]	-0.0397	0.1473	-0.2693	0.7889
HAM–D [points]	0.0174	0.1474	0.1177	0.9068
RMR [kcal/d]	-0.1966	0.1446	-1.3598	0.1805
Total physical activity [MET-min/day]	-0.3482	0.1382	-2.5189	0.0153
Total physical activity [counts/min]	-0.0771	0.1470	-0.5244	0.6025

HAM–D – Hamilton Depression Rating Scale; MoCA – Montreal Cognitive Assessment; RMR – Resting Metabolic Rate; SE – Standard Error

[1]Men *vs*. women

[2]City *vs*. village

[3]In a relationship *vs*. single

[4]Higher education *vs*. secondary + primary education

[5]Employed *vs*. unemployed

[6]At least good *vs*. lower than good

Table S14. Multivariate linear regression analysis assessing the relationship between MoCA points and selected variables in the NCF group (n = 48).

	β	SE	t	*p*
Age [years]	-0.1361	0.1346	-1.0108	0.3178
Place of living[2]	-0.2271	0.1341	-1.6940	0.0975
Family situation[3]	0.2214	0.1334	1.6604	0.1041
Total physical activity [MET-min/day]	-0.3106	0.1339	-2.3198	0.0252

HAM–D – Hamilton Depression Rating Scale; MoCA – Montreal Cognitive Assessment; RMR – Resting Metabolic Rate; SE – Standard Error

[1]Men *vs*. women

[2]City *vs*. village

[3]In a relationship *vs*. single

[4]Higher education *vs*. secondary + primary education

[5]Employed *vs*. unemployed

[6]At least good *vs*. lower than good
